# New Strategies of Canine Post-Adoption Support: Methods for a Prospective Longitudinal Cohort Study

**DOI:** 10.3390/ani15091232

**Published:** 2025-04-27

**Authors:** Emma L. Buckland, Kassandra Giragosian, Eleanor J. Jordan, Rosa E. P. Da Costa, Joshua L. Woodward, Rachel A. Casey

**Affiliations:** 1 Research, Dogs Trust, London EC1V 7RQ, UK; 2 School of Biological and Behavioural Sciences, Queen Mary University of London, London E1 4DQ, UK; k.giragosian@qmul.ac.uk

**Keywords:** post-adoption, behaviour, rehoming, health, longitudinal, relinquishment

## Abstract

In 2018, Dogs Trust (UK) launched the Post Adoption Support project, the largest known prospective cohort study investigating behavioural and health problems post-rehoming of dogs. Individuals adopting a dog from any UK Dogs Trust Rehoming Centre are eligible to receive phone calls regarding their dog’s health and behaviour at specific timepoints after adoption (2 days, 2 weeks, 4 months). Alongside providing an early and proactive framework to support the wellbeing of the adopter and dog, the project collects rich longitudinal data on dog health and behaviour following adoption to assess the development of issues, the impact of support given, and re-relinquishment risk. This paper details the methodology and study design of the Post Adoption Support project and describes a cohort profile of adoptions over an initial 6-month period. In the cohort, behaviour and health data were collected from 5498 adopters, with call success for those eligible ranging from 72% (4 months) to 89% (2 days). Data from these calls will provide significant insights into canine welfare during the first 4 months post-adoption, which can be used to inform strategies for effective and impactful rehoming and post-adoption support.

## Simple Summary

The Post Adoption Support project, initiated in 2018, aims to monitor the behaviour and health of dogs after they are adopted from Dogs Trust Rehoming Centres in the United Kingdom. Adopters receive phone calls at 2 days, 2 weeks, and 4 months post-adoption to check for any concerns about their dog’s health or behaviour and to be provided with appropriate relevant support, if requested. The project collects data over time to assess the occurrence of common health or behaviour problems, to evaluate the risk of returning the dog (re-relinquishment), and the impact of any support given. This study provides insights into adopted dog welfare during the first four months of adoption, which can be used to inform continuous improvement of processes for rehoming and post-adoption support.

## 1. Introduction

Globally, thousands of dogs are relinquished to rehoming organisations each year [1] to be adopted into new homes [2]. In the UK alone, the number of dogs relinquished each year is estimated to be approximately 130,000 [3], though the population is not formally monitored due to a lack of centralised reporting. One rehoming organisation (Dogs Trust) alone rehomed over 9700 dogs in 2022 [4]. In addition, estimates from a 2020 nationwide survey found that up to 12.1% of dogs in the UK were acquired from a rehoming charity website [5], suggesting that rehomed dogs make up a notable sub-section of the pet dog population.

The behaviour and health of pets after rehoming are often only informally monitored for a short period of time by rehoming organisations, and data are not routinely evaluated due to the scarce resources available. Most existing research has small sample sizes with fewer than a hundred dogs [6,7,8,9], and few studies have been able to follow dogs over several timepoints to evaluate changes over time (see exceptions in Neidhart and Boyd, 2002 [10] and Lord et al., 2007 [11]). Studies have also involved a range of different study designs (e.g., telephone/postal/in-person surveys and/or qualitative interviews) and/or definitions of reported behaviour issues (e.g., “bad habits” in Neidhart and Boyd, 2002 [10]; versus “pulling on the lead” in Marston et al., 2004 [6]). These methodological limitations hinder the application of the findings to real-world contexts. Additionally, these constraints do not allow for powerful statistical modelling that could effectively identify risk factors for re-relinquishment (return of the dog to the rehoming organisation) or the development of health or behaviour issues.

The frequency of reported health and behaviour issues is suggested to reduce over time post-rehoming, ranging from 72% of adopters reporting concerns one week after adopting [8], 68% reporting a concern one month after adopting [9,11], and 33% reporting concerns one year after adopting [10]. Undesirable behaviours are frequently reported as a reason for re-relinquishment, reaching as high as 60% in some studies (e.g., 59% [10]; 51% [12]). Specific behaviours frequently reported were aggression towards humans or other animals and/or destructive behaviour [13]. However, it is not known how many adopted dogs that stay in the home show these behaviours too, i.e., the true risk for dogs showing these behaviours. Compared to behaviour, fewer dogs are reported to be re-relinquished for health reasons (8% [14]), but health issues continue to be reported over 12 months post-adoption [11].

Evidence suggests that pre-emptive advice on behaviour provided before or at the time of adoption may have some benefit in preventing future issues [15]. However, to maximise the effectiveness of the advice provided, the gap in knowledge surrounding the risk of the development of behavioural issues over time must be addressed. By understanding common issues, their timeline, and the associated risk factors, rehoming organisations can better manage their resources. This will enable them to provide high-quality and effective support to prevent or alleviate these issues.

To tackle these knowledge gaps, in 2018, Dogs Trust (UK) launched a new initiative, named the Post Adoption Support project. This new initiative aimed to provide a more proactive, targeted support framework for adopters, which also allowed the collection of rich longitudinal data from owners on dog health and behaviour following adoption. The initiative involved increased telephone support and referral pathways, alongside data collection to evaluate effectiveness. The objective of the telephone calls is to proactively identify issues requiring support, improve adopter experience, and ultimately reduce the number of dogs being re-relinquished (returned) by offering free support and advice. The objective of the ongoing data collection is to identify commonly reported issues, including any changes over time and any associated risks, as well as to evaluate the impact of support on adoption success rates. The data from this longitudinal cohort initiative are being used to inform and guide best practices within rehoming organisations, leading to future dog and owner welfare benefits.

In this paper, we outline the methodology and study design of the Post Adoption Support research initiative. In addition, to demonstrate the value of this study, we describe a cohort profile of adoptions over a 6-month period in 2019, including the rates of opt-in to the support calls, response rates, and frequencies of health and behaviour issues described. We discuss the research questions that are intended to be answered using this cohort study and describe the longer-term studies that the project can contribute to.

## 2. Materials and Methods

### 2.1. Recruitment

The Post Adoption Support project was launched on 1 April 2018 for dogs rehomed to the public across all UK Dogs Trust Rehoming Centres. At the time of adoption, adopters are asked to sign a consent form for follow-up calls. A single post-adoption follow-up call, scheduled at a 2-day timepoint after rehoming, is offered as a default for all adopters as part of the terms and conditions of adoption (though it can be opted out of, if requested). Participants are also able to opt in to two additional calls, scheduled at a 2-week and 4-month timepoint post-adoption. This consent is stored digitally in a Customer Relationship Management (CRM) system (Salesforce), and consent is then re-confirmed at the start of each call. Participants can opt out of any call at any time.

The 2-day calls commenced on 1 April 2018, and the additional calls commenced on 23 August 2018 and 5 June 2019, respectively. Medical and behavioural ailments are treated during a dog’s stay at the Rehoming Centre, and all dogs are assessed by veterinary and behaviour professionals shortly before adoption (in addition to being routinely assessed during their stay). Any known issues are treated, and/or follow-up care is arranged with the new adopter. The collection and analysis of data from these post-adoption calls for research purposes was approved by an independent Ethical Review Board at Dogs Trust.

### 2.2. Procedure

Post-adoption follow-up calls are completed over the telephone by the Dogs Trust Customer Support Centre team during their standard opening hours. Adoption records and call records are stored in the CRM system and a list of scheduled calls is automatically generated each day for the respective call timepoint (2 days, 2 weeks, 4 months) from the date of rehoming, based on eligibility criteria where records show that owners have opted in, where the dog record indicates the dog remains in the home (i.e., not returned or deceased), and where there is a valid telephone number. Customer Support Centre staff follow a protocol for call completion, with a maximum of three attempts across three separate days, including one voicemail if applicable, before the call record is closed and marked unsuccessful. Customer Support Centre staff receive training and ongoing monitoring and support to deliver the calls and record the information in a standardised way.

### 2.3. Call Surveys

For each call, a standardised survey of scripted questions is used, and owner responses are recorded using single or multiple-choice categorical fields and/or free-text fields. A combination of closed- and open-ended question types is used. Using this combined approach, we aimed to capture both objective reporting of the common issues as evidenced in the literature and subjective reporting of any perceived issues. For closed questions, the question includes a list of pre-determined response options to capture objective reporting of pre-identified health conditions or behaviours, regardless of whether adopters have themselves perceived them as problematic. For open-ended questions, adopters are asked to describe any other issues they have seen to capture anything else that may be perceived as problematic by the adopter. For open-ended questions, pre-determined lists are available for the call agent to select from, based on the adopter responses, but these are not read out to adopters. For all question types, a free-text field “Other, please specify” is provided to capture responses outside of pre-determined lists. A summary of questions at each timepoint is provided below, and the full transcript of the questions at each timepoint is provided in Supplementary Materials.

**Figure 1:**
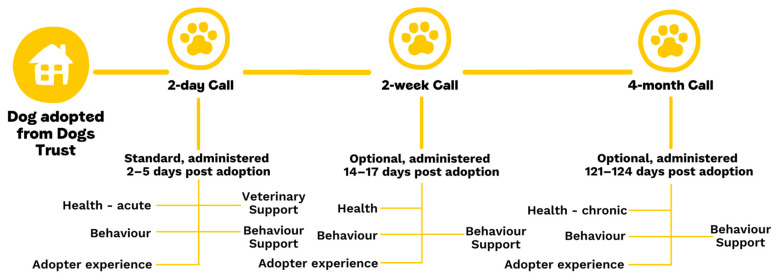
Description of the call timepoints and structure in the post-adoption study.

Calls are split into four sections—availability and consent, dog health, dog behaviour, and adopter experience of Dogs Trust (Figure 1). Data are recorded on call records directly in the CRM system.

### 2.4. Availability and Consent

Once successfully connected, adopters are greeted, and the purpose of the call is explained. They are asked a sequence of questions confirming that they are the named adopter on the record, that they still have the dog, and that they still want to proceed with the call. Call surveys are usually completed with the person named on the adoption record, but in some instances, surveys are also completed by a nominated contact who has previously given informed consent.

In addition, at 2 weeks and 4 months, a check is included to ask if the adopter has received health (2 weeks only) and/or behaviour support from Dogs Trust in the preceding 4 days. If so, the respective sections of the call are then skipped to prevent adopters from describing the issues for which they have already received support (data for which can be accessible through existing records).

### 2.5. Health

The health section differs across timepoints due to variations in prompted questions regarding the acute versus chronic nature of expected conditions following rehoming. At 2 days, adopters are asked a sequence of closed-ended questions relating to the presence and absence of vomiting, diarrhoea, coughing, lethargy, and balance since the time of adoption. At 4 months, closed-ended questions are used for the presence and absence of specific vomiting/diarrhoea and skin/coat issues within the last month. At all timepoints, adopters are asked to describe in their own words the presence and absence of any health issues that have arisen since adoption (2 days), the first few days of adoption (2 weeks), or in the last month (4 months). The reference to time here was used to minimise recall bias and avoid repetition of reporting across call timepoints. For this open-ended question, call agents use a pre-defined list of health issues to select from, but these are not read out to the adopter; other responses are collected as free-text.

Due to the nature of the script’s structure and data, and to allow comparison across questions and across timepoints, health issues were reclassified into groups for meaningful analysis. Code labels were chosen to reflect the descriptive text given by adopters whilst avoiding diagnostic terminology (e.g., ‘vomiting/diarrhoea’ was chosen instead of ‘gastroenteritis’) as the information was gathered by call centre staff without veterinary training, and dogs were not physically examined. The frequency and severity of a reported health issue were not asked, so categories included any mention of a particular health condition, regardless of frequency or severity.

### 2.6. Behaviour

The behaviour questions are the same across all three timepoints and are split into three distinct question sets, which group behaviours into ‘Potentially aggressive behaviours (PAB)’, ‘Separation-related behaviours (SRB)’, and ‘Other behaviours of concern (OB)’. The former two are closed-ended question types, and the latter is an open-ended question type. For all questions, adopters are asked to describe the presence of behaviours since adoption (2 days), excluding the first few days of adoption (2 weeks), or in the last month (4 months), respectively. The frequency and severity of a reported behaviour are not asked about, so categories included any mention of a particular behaviour regardless of frequency or severity.

#### 2.6.1. Q1: Potentially Aggressive Behaviours (PAB)

Adopters are asked if the dog has shown any of the listed behaviours (growling, baring teeth or wrinkling lips up, snapping, biting or nipping, lunging forward whilst barking, grabbing [e.g., grabbing lead], mouthing [e.g., at sleeves or arms], or standing very still and staring; Table 1). The list was devised from common agonistic-type canine behaviours, including low-level warning signals [16], and aimed to identify potential issues as early as possible to intervene and prevent escalation. If the presence of one or more behaviours was reported, the target (who/what the behaviour was directed towards) and situation (in what situation the behaviour occurred) were also recorded. For PABs, using the behaviour and target data collected, we further defined the data into two groupings (Table 1)—contact behaviour (PABc—biting or nipping, grabbing [e.g., grabbing lead], mouthing [e.g., at sleeves or arms] towards humans or other animals), and non-contact behaviour (PABnc—snapping, growling, baring teeth or wrinkling up lips, lunging forward whilst barking or standing very still and staring or PABc behaviour towards any other target [e.g., inanimate objects]). For the remainder of this paper, we describe PABc and PABnc within PAB, but we do not further describe detailed data on behaviour, target, or context.

#### 2.6.2. Q2: Separation-Related Behaviours

If adopters report the dog had been left home alone without human company, a follow-up question is asked to determine whether the dog had shown any of the following listed behaviours when they were left alone: barking, howling, whining or whimpering, destruction around the door, chewing or destruction of things other than his/her own toys, pacing, spinning, circling or tail chasing, panting when leaving, toileting, vomiting or drooling, excitability or excessive greeting, preventing owner leaving the room or trying to get out of the door with owner, or problems getting back into the house (Table 1).

#### 2.6.3. Q3: Other Behaviours of Concern

Finally, the adopter is asked if they have seen any other behaviours of concern (an open-ended question) and if so, would they like any support from Dogs Trust. This was chosen from a pre-determined list of behaviours (not read out to adopters), with an additional free-text option (see Table 1 for definitions of behaviours).

### 2.7. Support Offered

For questions on dog health and behaviour, pre-defined algorithms are embedded into the surveys such that specific responses trigger follow-up questions and/or prioritise an action for agents to offer a call-back from a specialist veterinary or behaviour team for advice on the response(s) given. Veterinary support is offered for specific acute conditions (e.g., vomiting/diarrhoea more than once, lethargy, coughing or breathlessness) reported during the first timepoint (2 days) only, and a call-back from a veterinary nurse is offered within 24 h. Beyond this timepoint, when health issues are reported, specific responses trigger the call agents to advise adopters to seek support from their own veterinary clinic. For behaviour, support is offered for any behaviour during all timepoints, and if requested, a call-back from a professional behaviour advisor is offered with varying timescales of escalation dependent on the potential severity of the issue—24 h (e.g., for aggression or separation-related behaviour), 48 h (e.g., for resource guarding, jumping up or toileting), or 72 h (e.g., for pulling on the lead, car travel, barking). When adopters are offered a call-back for support but do not wish to accept it, data are recorded as a binary variable (accepted/not), and reasons as to why, if provided, are recorded by staff in free-text sections.

### 2.8. Adopter Experience

In the final section of each call, adopters are asked questions regarding their experience and satisfaction with the adoption. At each timepoint, adopters are asked if they were happy to have adopted the dog, using a categorical response question. The calls included other questions regarding operational activities (e.g., feedback for rehoming, behaviour or training support received, or physical items given), but these are not reported further here.

### 2.9. Study Population

To describe the canine cohort profile, adoptions from 20 UK Dogs Trust Rehoming Centres over a 6-month period were analysed between 9 June and 9 December 2019. All data were extracted from Salesforce CRM. Dogs were followed for a minimum of 6 months post-adoption to determine if post-adoption calls were completed and if the dog remained in the home for this duration. Unique identifiers for the adopter, adoption, and dog were used to identify data from calls across multiple timepoints, where dogs were adopted twice (i.e., rehomed, returned, then rehomed) or if adopters had adopted multiple dogs during the study period.

Dog demographic data were described using breed (single-breed, mixed-breed, or unknown), age (calculated from known or estimated date of birth), and sex. The age variable was converted into categorical bins for analysis, comprising Puppy (0–6 months), Adolescent (6–24 months), Mature Adult (2–6 years), and Senior/Geriatric (6+ years) groups, as per classification in Harvey et al., 2021 [17]. In addition, the Rehoming Centre or specialist rehabilitation unit adopted from, whether the dog was fostered or in kennels, the date of homing, and the partial postcode of their new home were also recorded, along with the date and reason for return (where applicable).

### 2.10. Preparing the Dataset for Analysis

Data were stored in the CRM system and extracted for analysis using existing reporting capabilities within Salesforce. Adoption records, call records, and call survey data were extracted, and data were exported to Microsoft Excel and stitched together using unique adoption identifiers. The dataset was analysed using R v4.2.1 [18] via RStudio^®^ v2022.7.2.576.

#### 2.10.1. Exclusions

Adoptions were excluded from further analysis if adopters did not opt in to post-adoption support (2-day call) or did not provide a valid telephone number at the time of adoption. Additionally, adoptions were excluded if the adoption or call records failed to load (i.e., system errors) and, therefore, calls were not scheduled. Further, though they took place, calls were excluded from this analysis for adopters with multiple dogs adopted on the same day (as these calls took a different format to capture details about both dogs) and for dogs that were adopted by their foster carer (as this would deem the timepoints inapplicable). Where adopters rehomed more than one dog during the study period, but not at the same time, only the first adoption (primary adoption) was analysed to avoid clustering effects of the household.

#### 2.10.2. Cohort Data

Geocoordinates for each outward partial postcode were obtained and used to map the approximate location of adoptions using the R packages *ggplot2* version 3.5.1 [19] and *maps* version 3.4.2 [20]. Digital vector boundaries for “Counties and Unitary Authorities” [21] and “Regions” [22] in the UK were obtained and used to count the number of adoptions within each county by transforming the geocoordinates of the partial postcodes into digital vector boundaries with the R package *sf* [23,24]. London boroughs and unitary authorities within the region of London were summarised as the county of Greater London.

#### 2.10.3. Coding and Grouping of Reported Issues

To prepare the dataset for analysis, all free-text entries for every ‘Other, please specify’ response for behaviour and health questions were coded and were either allocated into an existing response from the pre-defined list or a newly created response for each question. Health questions were coded by one author (RDC), and behaviour questions were coded by three authors (JW, EB, KG)—inter-rater reliability was therefore investigated for behaviour coding using the Kappa statistic.

Responses were reviewed by authors (EB, KG, EJ) and grouped to avoid overlapping or ambiguous categories and to combine those with insufficient values for meaningful analysis. Where appropriate, grouping categories were validated by professional veterinary and behaviour colleagues within Dogs Trust.

## 3. Results

### 3.1. Cohort Profile

During the study period, 6196 adoptions took place from 20 UK Dogs Trust Rehoming Centres. There were 574 (9.3%) adoptions of multiple dogs—562 (9.1%) rehomed two dogs and 12 (0.2%) rehomed three dogs during the study period, with most (507, 87.0%) adopting the dogs at the same time.

A total of 629 (10.2%) adoption records were excluded from further analysis of call records—86 due to bad data (invalid telephone number, missing call records) and 543 due to multi-dog adoption households (same-day or non-primary adoptions). With these exclusions, we therefore describe a total of 5567 adoptions in this cohort, comprising 5567 unique adopters and 5194 unique dogs (as some dogs were adopted, returned, and then re-adopted by a new owner).

### 3.2. Dogs Returning to Kennel and Opt-In Rates

Regardless of the opt-in rate for the calls, 4757 (85.4%) dogs remained in the home at 6 months post-adoption (Table 2). Overall, the opt-in rate for the 2-day call was 98.8% (5498) of adoptions. For 2 weeks/4 months, the opt-in rate was 83.8% (4663). However, as detailed in Table 2, for 92 (1.7%) adoptions, a return occurred within 0–2 days; so for these adoptions, no calls were attempted irrespective of the adopters’ opt-in status.

The following results are based on data from 5498 participants who opted into this study.

### 3.3. Adopter Location and Dog Demographics

**Figure 2:**
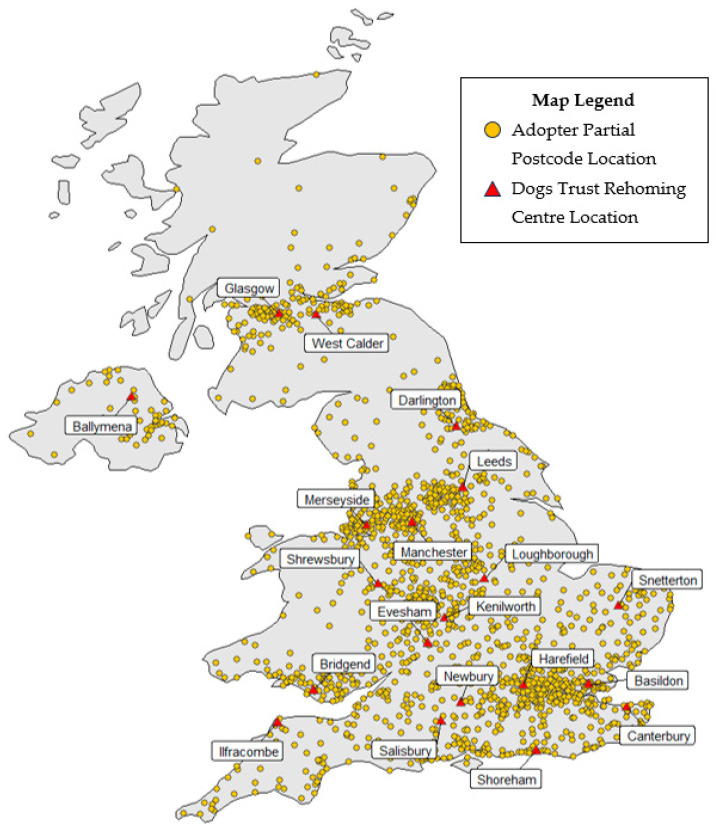
Geographical distributions of adopters (*n* = 5495) and Dogs Trust Rehoming Centre locations (*n* = 20) in the UK.

Adoptions with valid partial postcodes (*n* = 5495) were used to create a geographical map (Figure 2) with adopter and Dogs Trust Rehoming Centre locations. The majority of adopters resided in England (84.0%, 4614; Table 3).

The majority of adoptions (88.9%; 4890) placed the dogs from standard kennels within Rehoming Centres, whereas 23 (0.5%) were adopted from kennels at specialist rehabilitation units located within the Dogs Trust Centres, and 585 (10.6%) were adopted from a foster home scheme.

Dog sex was slightly skewed towards males (3140, 57.1%) compared to females (2358, 42.9%). Over 60% of adoptions were mixed-breeds (3401, 61.9%) compared to single-breeds (2048, 37.2%); the breed was unrecorded for 49 adoptions (0.9%).

Most dogs were mature adults (2192, 39.9%), adolescents (1424, 25.9%), or seniors (1022, 18.6%). Puppies (634, 11.5%) and geriatric dogs (226, 4.1%) were much less common.

### 3.4. Call Success

There were 5412 adoptions who were eligible to receive a 2-day call. Of these adoptions, 4799 (88.7%) 2-day calls were successfully completed. Calls were not attempted for 21 (0.4%) dogs in the process of being returned (as seen within notes in the CRM system). Of those who did not complete the call, the majority (548; 92.6%) were not contactable and were not reached within three call attempts, but for 44 (7.4%), the call was not progressed due to not wanting to complete the call (35) or no consenting adopter being available (9).

There were 4346 adoptions who were eligible to receive the 2-week calls. Of those adoptions, 3554 (81.8%) 2-week calls were successfully completed. Calls were not attempted for 34 (0.8%) dogs in the process of being returned. Of those who did not complete the call, the majority (686; 90.4%) of adopters were not contactable, but for 73 (9.6%), the call was not progressed due to not wanting to complete the call (66) or no consenting adopter being available (7).

There were 4018 adoptions who were eligible to receive the 4-month calls. Of those adoptions, 2889 (71.9%) 4-month calls were successfully completed. Calls were not attempted for seven (0.2%) dogs in the process of being returned. Of those who did not complete the call, the majority (973; 86.7%) of adopters were not contactable, but for 149 (13.3%) the call was not progressed due to not wanting to complete the call (130), no consenting adopter being available (11) or the adopter mentioning the dog was deceased (4) or passed to a different home (4).

### 3.5. Health Issues

A total of 1902 free-text entries for health questions were coded and collapsed into pre-existing or new categories.

Of those who completed the health section, the presence of at least one health-related issue was reported by 56.1% (2692) of adopters at 2 days, 16.7% (588) at 2 weeks, and 38.8% (112) at 4 months (Table 4). The most reported health-related issue discussed at all three timepoints was vomiting/diarrhoea (Table 4).

### 3.6. Behaviour Issues

A total of 1667 free-text entries for behaviour questions were coded, from across the timepoints, into categorical variables and collapsed into pre-existing or new categories for meaningful analysis. Inter-rater reliability showed substantial agreement between coders for PAB (κ = 0.83, *p* < 0.001), moderate agreement for separation-related behaviours (κ = 0.538, *p* = 0.002), and near-perfect agreement for other behaviours of concern (κ = 0.819, *p* < 0.001).

Of those who completed the behaviour section, the presence of at least one behaviour-related issue was reported by 54.0% (2590) of adopters at 2 days, 60.6% (2100) at 2 weeks, and 57.4% (1635) at 4 months (Table 5).

The presence of PABs was reported by more than a third of adopters at each timepoint, though proportionally, contact PABs were reported less commonly than non-contact PABs (18.0% versus 28.0%; Table 5).

The number of adopters who reported that they had left their dog alone increased with time since adoption (2 days: 32.7%, 1570; 2 weeks: 72.1%, 2499; 4 months: 86.1%, 2452), while for dogs who were left alone, the reported presence of separation-related behaviours decreased over time (2 days: 37.3%, 585; 2 weeks: 19.0%, 475; 4 months: 15.0%, 367).

The presence of other behaviours of concern remained consistent (between 21 and 24%) across timepoints (Table 5). The three most common other behaviours of concern at all timepoints were issues on the lead, vocalisations, and resource-related behaviours (Table 5).

### 3.7. Support Offered

Of those who completed the 2-day call, 60.3% (2892/4799) were offered a callback for further support—72.5% (2097) were for behaviour support, 11.4% (330) were for health support, and 16.1% (465) were for both behaviour and health support. Of those who were offered a callback for behaviour support (2562), 81.9% (2097) were for 24 h, 7.4% (190) for 48 h, and 10.7% (275) for 72 h callback timeframes.

Of those who completed the 2-week call, 55.7% (1979/3554) were offered a callback for further behaviour support—83.9% (1661) were for 24 h, 5.5% (109) were for 48 h, and 10.6% (209) were for 72 h timeframes, respectively.

Of those who completed the 4-month call, 52.0% (1502/2889) were offered a callback for support—83.4% (1252) were for 24 h, 6.6% (99) were for 48 h, and 10.1% (151) were for 72 h timeframes, respectively.

### 3.8. Adopter Experience

At 2 days, 97.7% (4690) adopters were happy to have adopted their dog, and this increased to 98.5% (3500) at 2 weeks and 98.9% (2859) at 4 months. At each timepoint, less than 2% of adopters reported “not sure as it depends on his/her behaviour and/or health” or “not sure, it was too early to say” and less than 1% selected “no, unhappy about the adoption”. The adoption experience was largely rated as “very positive” or “positive” at both 2 days and 4 months, respectively (2 days: very positive: 4057, 84.5%; positive: 611, 12.7%; 4 months: very positive: 2416, 83.6%; positive: 371, 12.8%).

## 4. Discussion

This paper describes the methodology and an example dataset from the largest, to our knowledge, longitudinal cohort study of adopted dogs worldwide. This was possible due to the integral nature of this study being embedded within the adoption process at the largest dog rehoming organisations in the UK. Importantly, the project will continue to provide data on adopted dog behaviour and health as it also provides proactive support to adopters, where required, during their adoptive journey. The longitudinal and structured methodology offers a unique opportunity to provide meaningful analysis of health and behaviour outcomes (and the combination of these) that are present shortly after the point of adoption (2 days) or that develop over time in the home (2 weeks or 4 months). This approach also allows for an investigation of the risk of return and evaluation of the impact of support provided by the rehoming organisation. The development of health and behaviour issues in adopted dogs, and indeed the general canine population, is not well studied, especially not concurrently or over multiple timepoints. Research has been limited in these areas due to the use of cross-sectional design [11] or small sample sizes [25]. Our study cannot ascertain finer details of the reported presence of issues, such as severity, frequency, or clinical diagnosis, due to the owner-reported nature of the data, but also due to the wide-ranging and broad categories used for analysis (such as ‘appetite change’), which limits specificity. Indeed, the perception of an issue does not indicate abnormalities, since some reported behaviours may be within the range of normal canine behaviour that can be perceived as problematic or unwanted for an owner (e.g., toileting on an unwanted area of the garden [26]), yet they warrant support for owners to alleviate or manage their preferences. This is reflected in the proportional nature of data, which should be interpreted with less sensitivity or specificity than other validated questionnaires for identifying specific health or behaviour issues (e.g., [27]). While owner-reported data must be treated with caution and cannot be interpreted as clinical diagnoses, it can be useful for the early identification of issues and understanding of the owner’s perception of issues.

The opt-in and completion rates for the project were notably higher in comparison to other longitudinal studies (e.g., 71–47% [28]), with an expected decline over time of 89% at 2 days, 82% at 2 weeks, and 72% at 4 months. Notably, the number of adopters who were reached but did not want to complete the call increased at the 4-month timepoint. This could be related to the sizeable gap between the 2-week and 4-month call; with the increasing length of time since adoption, owners may have felt support was no longer needed. Telephone calls have been reported to provide higher response rates than traditional paper questionnaires [29]. Such high opt-in and completion rates are indicators that our study population is likely to be a good representation of rehomed dogs across the rehoming organisation, and perhaps across the rehomed population in the UK, given the large and national distribution of Dogs Trust Rehoming Centres (Figure 2). In a comparable study using telephone calls in Melbourne, Australia, Marston and colleagues calculated that their sample size represented just 10% of all adoptions during the study period [6]. Representation at a population level is difficult to ascertain since both the number of owned dogs and the number of dogs rehomed by organisations are not centrally recorded in the UK, though the UK dog population was estimated to be 9.9 million in 2019 [30]. It is recognised, however, that the cohort is likely to suffer from self-selection sampling bias in those adopters choosing to complete our surveys versus those who choose not to. Indeed, responses were exceptionally skewed towards the positive end of the scale regarding the adoption experience and satisfaction in adopting the dog, indicating a possible selection bias—but perhaps also suggesting a lack of sensitivity and specificity of the scale used. Our study is also likely to be affected by survivorship bias, since we cannot ascertain the health and behaviour issues of dogs who were returned or those who did not opt in or were not successfully contacted. In addition, the cohort study does not collect any demographics of adopters, other than postcode, thus we cannot be certain that our cohort best represents the dog-owning population, with other variables such as owner age, gender, and household information, though these are also poorly understood in the wider population. Other longitudinal studies where demographics were collected have shown participatory bias based on gender, age, and education status [28], and it is expected that these biases may impact exposure and outcome measures intended for the PAS project, and therefore the generalisability of our findings. However, the effect of such sample biases can be reduced when we value the cohort itself, using motivated and engaged participants, over the representativeness of the cohort [31].

The rehoming of dogs is an ever-changing landscape, with Dogs Trust recording an 11% increase over 4 years in dogs handed to them due to owner relinquishment compared to those handed in as strays or through other organisations [4,32,33,34,35]. Our study offers a unique opportunity to investigate the changes in the rehoming sector over time, by monitoring the nature and frequency of behaviour and health issues experienced in dogs from adoption and over time. In addition, when linked with other operational data, it can also be used to examine the predictive validity of owner-reported information and welfare assessments on post-adoption outcomes [36]. For example, in a small study spanning 6 weeks post-adoption, relinquishing owner reports correlated to adoptive owner reports in only 9 of 20 behaviours measured [29]. Identifying trends in reported behaviour and health issues, especially those linked to re-relinquishment, can aid the design of tailored interventions before adoption, such as behavioural modification plans, which may reduce the risk of problems in the home. Indeed, the Post Adoption Support project also offers an opportunity to evaluate the impact of those interventions.

The Post Adoption Support project aimed to follow dogs at multiple timepoints, with a particular emphasis on identifying early signs of potential issues before escalation or establishment, to offer proactive support and to reduce re-relinquishment. Re-relinquishment is sometimes referred to as a measure of failed adoption [13], but the provision of support could also influence the rate of return, for example, offering increased opportunities for discussing the return of the dog. The overall re-relinquishment rate in our study was 14.6%, which, although consistent with a study using an earlier sample of adoptions from Dogs Trust Rehoming Centres [13], may be a more representative proportion due to the larger sample size. In addition, these studies may also not be comparable due to populational changes in dogs and operational changes in rehoming over time. Several studies have investigated the risk of return associated with using dog and owner demographics, using retrospective data (e.g., [7,37,38]). However, few studies have been able to evaluate the success of pre-adoption interventions to mitigate these risks. Our study offers an opportunity to do this by following dogs prospectively through adoption and post-adoption. In this way, it is hoped to provide more exploration of the nuanced measures of adoption success and the risk of return, such as whether adopters accepted the support when reporting issues.

### 4.1. Health Issues

Overall, we found a decrease in the reported presence of health-related concerns over time (56% at 2 days to 39% at 4 months). The higher frequency during early adoption may reflect the abrupt change in environment for the dogs as they move into their new homes [39], though it may also be explained by more verbal prompts for specific health issues in the earliest call. This is perhaps also seen in the increased proportion of reported presence of skin issues in our study over time, since these are verbally prompted during the 4-month call, though it may also be explained that chronic issues may develop over time as dogs age, and this requires further analysis. Among the few studies where post-adoption veterinary access was recorded, most adopters accessed veterinary care for their dog in the first month of adoption [12,40], and in the first 12 months of adoption, visits to a veterinarian were common [10]. The general decrease in the presence of health concerns over time is also reflected in previous studies [8,11], as is the finding that the presence of vomiting/diarrhoea was the most common health issue reported across all timepoints [41]. These data can be particularly useful in raising awareness of expected problems—to both help minimise them and set new adopter expectations, especially for when to seek veterinary advice for more serious problems [41]. In general, the prevalence of such health issues at a population level is not attainable since data only exists for those attending clinical care [42].

### 4.2. Behaviour Issues

Over 50% of adopters reported the presence of at least one behaviour concern at each timepoint, and this is comparable to previous estimates, which typically ranged from 60 to 70% at 1-month post-adoption [9,11,25]. Estimates in the general pet dog population have suggested that up to 80% of dogs exhibit behaviours that owners find problematic [43,44]. However, differences in study profile and design may play a role in prevalence estimates; thus, studying proportional changes over time within a single population will offer valuable insights. Both aggressive-related behaviours and SRB are commonly reported as problematic by dog owners [45,46], and in this project, we specifically asked about the presence of these behaviours using a prompted question at each timepoint; thus, we expect the reported frequency of these issues to be higher. We asked about a broad range of behaviours that could be considered potentially aggressive (PAB; since we do not infer motivation) in any context, and less than half of adopters reported the presence of this type of behaviour at each timepoint, but reporting did appear to increase over time (2 days: 36%; 2 weeks: 44%; 4 months: 42%). In previous work, aggression has been more specifically defined (e.g., towards people), and its prevalence appears lower within ranges of 10–25% [12,25,47]. Our study also offers the opportunity to analyse specific behaviours, targets, and contexts surrounding any aggression-related behaviour. For example, many of these behaviours occurred towards the owner or an adult household member or towards an unfamiliar dog, and the behaviour was reported to occur during play or when the dog was reacting to another animal. Future work can help to identify patterns over time for key behaviours, targets, and contexts where aggression-related behaviour might occur and those that are linked to re-relinquishment.

As with other behaviours, prevalence estimates for SRB varied in other post-adoption cohorts, from 14% [48] to 43% [49]. In our study, we found that 37% of dogs left alone were reported to show SRB at 2 days after adoption, falling to 15% of dogs who were left alone at 4 months. Conversely, SRB has been reported to increase over time (14% at 1 week increasing to 28% at 12 weeks [50], perhaps due to a greater likelihood of being left alone for longer periods. Due to the difficulties of identification and perception, SRB are thought to be underestimated, especially when reported unprompted [51]. We found very low instances of the presence of other behaviours of concern, within the unprompted question, with the presence of all other behaviour concerns reported by less than 5% of adopters. The most reported behaviours of concern are in line with those found in other research for adoption dog populations, for example, toileting issues and inappropriate vocalisations [10,41]. Using our data, patterns of the presence of these reported behaviours over time, including animal-related factors such as age or gender, can be further explored in addition to advice-seeking and re-relinquishment patterns linked to key behaviours.

## 5. Conclusions

To our knowledge, the post-adoption study project as described in this paper is the largest prospective cohort project on the success of canine adoptions and on the development of behavioural and health issues post-rehoming, with healthy completion rates enabling us to provide representative data during the first four months of adoption for the UK population of adopted dogs. We acknowledge likely differences in selection criteria and processes across rehoming organisations as well as the self-selection bias of participating adopters. Nonetheless, our project benefits from a prospective and longitudinal study design which allows for powerful modelling, reduces the impact of recall bias, and enables evaluation of temporal effects. We hope our findings on the frequency of reported health and behaviour issues post-adoption—along with those from future projects within the project—will provide evidence-based insights for dog welfare and effective and impactful post-adoption support in rehoming organisations worldwide.

## Figures and Tables

**Table 1:** Within the behaviour question section (Q1–3), a list of the behaviour categories within the dataset, and examples of the corresponding descriptors used to define them. Descriptors were collated from the responses within both the pre-defined list of behaviours and the free-text “other” response.

Behaviour Category		Descriptors
Q1: Potentially aggressive behaviour (PAB)	Contact (PABc)	Biting or nipping, grabbing [e.g., grabbing lead], mouthing [e.g., at sleeves or arms] ***towards*** humans (owner or adult/child member of household, adult/child visitor to the house, adult/child when outside of the house) or other animals [unfamiliar/familiar dog; household cat, other pets, livestock or horses, wildlife or local cats).
	Non-contact (PABnc)	Snapping, growling, baring teeth or wrinkling up lips, lunging forward whilst barking or standing very still and staring or contact behaviours ***towards*** any target and contact behaviours [biting or nipping, grabbing, mouthing] towards any other target (inanimate object, noise or sound, flying insect).
Q2: Separation-related behaviour (SRB) (behaviour is presented only when owner is not present)		Barking; howling; whining or whimpering; toileting; destruction or displacement of things other than his/her own toys; spinning or tail chasing; vomiting; drooling or panting as owner left or returned; trying to stop owner leaving the room or trying to get out of the door with owner; excitability or excessive greeting when owner returned; chewing or scratching around doorway or windows; described as stressed, anxious or upset; escaping; fixates on door; overactive/does not settle.
Q3: Resource behaviour		Chews or destroys items; digging/scratching surfaces; food guarding; steals, scavenges, snatches or begs food; goes/will not go places owner does not want; escaping; will not get off sofa/other furniture; steals items or will not give them up; buries/caches items.
Q3: On/off lead issues		Chasing; runs up to people or dogs off lead; does not come back when called; lead chewing/biting/ragging; refuses to put on harness/lead; difficult to walk on the lead, e.g., pulling on the lead or refuses to walk.
Q3: Toileting issues		Toileting inside the home; toileting issue outside of the home.
Q3: Vocalisations		Barking (at people or animals/objects/excessively); vocalisation (unspecified); whining (with owners present).
Q3: Issues with people		Attached to someone in the household; pawing at or licking people; follows people around house and/or trips people up; jumping up; shies away from/wary of people/men/visitors/strangers; unable to be handled/groomed
Q3: Anxiety/avoidance (general)		Anxiety; shaking; pacing; panting; wary; runs away/hides (unspecified/from moving vehicles); reluctant to go in/out of places; reluctant to walk/worried on walks.
Q3: Excitable/unsettled		Excitable; wants to play excessively; vocalisations/unsettled behaviour—overnight; overactive/does not settle down.
Q3: Abnormal behaviour		Excessive grooming or self-injury; fixates/stares (unspecified/inanimate/animals/people); spins in circles or chases tail repetitively; licking/sucking inanimate objects; coprophagia; pica (organic/inorganic); unresponsive/uninterested in play.
Q3: Mounting/humping		Mounts/humps person, dog, or object (e.g., bed)
Q3: Issues with other dogs		Reacts to other dogs on walks e.g., squeals, fixates, submissive, over-friendly; bothers a household/non-household dog; excited with other dogs; plays excessively or roughly with another dog; wary of/runs away/hides from other dogs.

**Table 2:** Return rates within the cohort, according to the level of consent for post-adoption support.

Time in the Home Post-Adoption	Adoptions n (%)	Did Not Opt-in n (%)	Opt-in to 2-Day Call Only n (%)	Opt-in to All Calls n (%)
Remained in home at 6 months	4757 (85.4%)	57 (82.6%)	738 (88.4%)	3962 (85.0%)
0–2 days	92 (1.7%)	2 (2.9%)	12 (1.4%)	78 (1.7%)
3–14 days	284 (5.1%)	5 (7.3%)	29 (3.5%)	250 (5.3%)
15–121 days	375 (6.7%)	4 (5.8%)	47 (5.6%)	324 (6.9%)
122–182 days	59 (1.1%)	1 (1.55%)	9 (1.1%)	49 (1.1%)
Total	5567 (100%)	69 (100%)	835 (100%)	4663 (100%)

**Table 3:** The number and proportion of adopters (*n* = 5495) across the countries in the UK and across the top 10 counties in the UK.

**Country**	***n***	**%**
England	4614	84.0
Scotland	396	7.2
Wales	292	5.3
Northern Ireland	193	3.5
**County**	***n***	**%**
Essex	198	3.6
Greater London	187	3.4
Warwickshire	181	3.3
Shropshire	172	3.1
Kent	168	3.1
Worcestershire	160	2.9
Hampshire	121	2.2
Leeds	120	2.2
Leicestershire	119	2.2
Devon	107	1.9

**Table 4:** The number and proportion of adopters reporting the presence of health issues at each timepoint, of those who completed the health section of the call (in bold). Only the five most commonly reported specific health issues (out of 21 categories) are displayed. Adopters could report the presence of multiple health issues, so proportions will not add up to 100%.

		Number (%) 2 Days	Number (%) 2 Weeks	Number (%) 4 Months
Number of successful calls		4799	3554	2889
**Adopters who completed the health section**		**4795** **(99.9)**	**3529** **(99.3)**	**2883** **(99.8)**
Adopters who reported at least one health issue		2692 (56.1)	588 (16.7)	1120 (38.8)
Health topics reported	Vomiting/diarrhoea	1659	212	718
		(34.6)	(6.0)	(24.9)
	Respiratory issue	948	154	49
		(19.8)	(4.4)	(1.7)
	Skin issue	195	72	304
		(4.1)	(2.0)	(10.5)
	Appetite change	426	24	4
		(8.9)	(0.7)	(0.1)
	Eyes/ears/nose/mouth issue	105	75	140
		(2.2)	(2.1)	(4.9)

**Table 5:** The number and proportion of adopters reporting the presence of behaviour issues at each timepoint, of those who completed the behaviour section of the call (in bold). For Other Behaviour (OB), only the ten most commonly reported behaviours are displayed (out of 14). Adopters could report the presence of multiple behaviours, so proportions will not add up to 100%.

		Number (%) 2 Days	Number (%) 2 Weeks	Number (%) 4 Months
Number of successful calls		4799	3554	
**Adopters who completed the behaviour section**		**4799** **(100)**	**3468** **(97.6)**	**2847** **(98.6)**
Adopters who reported at least one behaviour issue		2590	2100	1635
		(54.0)	(60.6)	(57.4)
Behaviours reported	Potentially aggressive behaviours	1738	1525	1205
		(36.2)	(44.0)	(42.3)
	Contact behaviour	766	737	500
		(16.0)	(21.3)	(17.6)
	Non-contact behaviour	1201	1017	892
		(25.0)	(29.3)	(31.3)
	Separation related behaviours *	585	475	367
		(37.3)	(19.0)	(15.0)
	Other Behaviours (total)	1104	830	607
		(23.0)	(23.9)	(21.3)
	On/off lead issues	181	172	170
		(3.8)	(5.0)	(6.0)
	Vocalisations	156	124	130
		(3.3)	(3.6)	(4.6)
	Resource-related behaviour	193	129	64
		(4.0)	(3.7)	(2.3)
	Toileting issues	160	108	66
		(3.3)	(3.1)	(2.3)
	Issues with people	136	75	59
		(2.8)	(2.2)	(2.1)
	Anxiety/avoidance	104	69	41
		(2.2)	(2.0)	(1.4)
	Issues with other dogs	51	58	39
		(1.1)	(1.7)	(1.4)
	Abnormal behaviour	62	48	33
		(1.3)	(1.4)	(1.2)
	Excitable/unsettled	68	41	26
		(1.4)	(1.2)	(0.9)
	Travel-related issues	41	31	33
		(0.9)	(0.9)	(1.2)
Health and Behaviour	Adopters who completed both health and behaviour sections	4795	3442	2842
		(99.9)	(96.8)	(98.4)
	Adopters reporting both health and behaviour	1580	390	752
		(32.9)	(11.3)	(26.0)

* % is out of only those left alone (2 days = 1570, 2 weeks = 2499, 4 months = 2452, total = 6521).

## Data Availability

The data generated and analysed during the study are not publicly available, but anonymised versions can be made available from the corresponding author upon reasonable request.

## Supplementary Materials

The following supporting information can be downloaded at: https://www.mdpi.com/article/10.3390/ani15091232/s1, A full transcript of questions, responses and logic (action) for each call script at 2-day, 2-week and 4-month timepoints, respectively.

## References

[1] Coe J.B., Young I., Lambert K., Dysart L., Nogueira Borden L., Rajić A. (2014). A Scoping Review of Published Research on the Relinquishment of Companion Animals. J. Appl. Anim. Welf. Sci..

[2] Weiss E., Gramann S., Victor Spain C., Slater M. (2015). Goodbye to a Good Friend: An Exploration of the Re-Homing of Cats and Dogs in the U.S. Open J. Anim. Sci..

[3] Clark C.C., Gruffydd-Jones T., Murray J.K. (2012). Number of Cats and Dogs in UK Welfare Organisations. Vet. Rec..

[4] Dogs Trust Annual Report 2021. https://www.dogstrust.org.uk/downloads/annual-report-2021.pdf.

[5] Anderson K.L., Casey R.A., Cooper B., Upjohn M.M., Christley R.M. (2023). National Dog Survey: Describing UK Dog and Ownership Demographics. Animals.

[6] Marston L.C., Bennett P.C., Coleman G.J. (2004). What Happens to Shelter Dogs? An Analysis of Data for 1 Year From Three Australian Shelters. J. Appl. Anim. Welf. Sci..

[7] Shore E.R. (2005). Returning a Recently Adopted Companion Animal: Adopters’ Reasons for and Reactions to the Failed Adoption Experience. J. Appl. Anim. Welf. Sci..

[8] Vitulová S., Voslářová E., Večerek V., Bedáňová I. (2018). Behaviour of Dogs Adopted from an Animal Shelter. Acta Vet. Brno.

[9] Wells D.L., Hepper P.G. (2000). Prevalence of Behaviour Problems Reported by Owners of Dogs Purchased from an Animal Rescue Shelter. Appl. Anim. Behav. Sci..

[10] Neidhart L., Boyd R. (2002). Companion Animal Adoption Study. J. Appl. Anim. Welf. Sci..

[11] Lord L.K., Yaissle J.E., Marin L., Couto C.G. (2007). Results of a Web-Based Health Survey of Retired Racing Greyhounds. J. Vet. Intern. Med..

[12] Lord L., Reider L., Herron M.E., Graszak K. (2008). Health and Behavior Problems in Dogs and Cats One Week and One Month after Adoption from Animal Shelters. J. Am. Vet. Med. Assoc..

[13] Diesel G., Pfeiffer D.U., Brodbelt D. (2008). Factors Affecting the Success of Rehoming Dogs in the UK during 2005. Prev. Vet. Med..

[14] Hawes S.M., Kerrigan J.M., Hupe T., Morris K.N. (2020). Factors Informing the Return of Adopted Dogs and Cats to an Animal Shelter. Animals.

[15] Blackwell E.J., Casey R.A., Bradshaw J.W.S. (2016). Efficacy of Written Behavioral Advice for Separation-Related Behavior Problems in Dogs Newly Adopted from a Rehoming Center. J. Vet. Behav..

[16] Lockwood R. (2016). Ethology, Ecology and Epidemiology of Canine Aggression. The Domestic Dog: Its Evolution, Behaviour & Interactions with People.

[17] Harvey N.D. (2021). How Old Is My Dog? Identification of Rational Age Groupings in Pet Dogs Based Upon Normative Age-Linked Processes. Front. Vet. Sci..

[18] R Core Team R: A Language and Environment for Statistical Computing; R Foundation for Statistical Computing 2022, Vienna, Austria. https://www.R-project.org/.

[19] Wickham H. Ggplot2: Elegant Graphics for Data Analysis 2016. https://ggplot2.tidyverse.org.

[20] Becker R., Wilks A. Maps: Draw Geographical Maps 2023. https://CRAN.R-project.org/package=maps.

[21] Office for National Statistics Counties and Unitary Authorities (December 2023) Boundaries UK BFC. https://geoportal.statistics.gov.uk/datasets/ons::counties-and-unitary-authorities-december-2023-boundaries-uk-bfc-2/about.

[22] Office for National Statistics Regions (December 2023) Boundaries EN BFC. https://geoportal.statistics.gov.uk/datasets/ons::regions-december-2023-boundaries-en-bfc-2/about.

[23] Pebesma E. (2018). Simple Features for R: Standardized Support for Spatial Vector Data. R J..

[24] Pebesma E., Bivand R. (2023). Spatial Data Science: With Applications in R.

[25] Gates M.C., Zito S., Thomas J., Dale A. (2018). Post-Adoption Problem Behaviours in Adolescent and Adult Dogs Rehomed through a New Zealand Animal Shelter. Animals.

[26] Pirrone F., Pierantoni L., Mazzola S.M., Vigo D., Albertini M. (2015). Owner and Animal Factors Predict the Incidence of, and Owner Reaction toward, Problematic Behaviors in Companion Dogs. J. Vet. Behav..

[27] Brady K., Hewison L., Wright H., Zulch H., Cracknell N., Mills D. (2018). A Spatial Discounting Test to Assess Impulsivity in Dogs. Appl. Anim. Behav. Sci..

[28] Murray J.K., Kinsman R.H., Lord M.S., Da Costa R.E.P., Woodward J.L., Owczarczak-Garstecka S.C., Tasker S., Knowles T.G., Casey R.A. (2021). “Generation Pup”–Protocol for a Longitudinal Study of Dog Behaviour and Health. BMC Vet. Res..

[29] Stephen J., Ledger R. (2007). Relinquishing Dog Owners’ Ability to Predict Behavioural Problems in Shelter Dogs Post Adoption. Appl. Anim. Behav. Sci..

[30] PDSA PAW Animal Wellbeing Report 2019. https://www.pdsa.org.uk/what-we-do/pdsa-animal-wellbeing-report/past-reports.

[31] Nohr E.A., Liew Z. (2018). How to Investigate and Adjust for Selection Bias in Cohort Studies. Acta Obs. Gynecol. Scand..

[32] Dogs Trust Annual Report 2017. https://www.dogstrust.org.uk/downloads/annual-report-2017.pdf.

[33] Dogs Trust Annual Report 2018. https://www.dogstrust.org.uk/downloads/annual-report-2018.pdf.

[34] Dogs Trust Annual Report 2019. https://www.dogstrust.org.uk/downloads/annual-report-2019.pdf.

[35] Dogs Trust Annual Report 2020. https://www.dogstrust.org.uk/downloads/annual-report-2020.pdf.

[36] Clay L., Paterson M., Bennett P., Perry G., Rohlf V., Phillips C.J.C. (2020). In Defense of Canine Behavioral Assessments in Shelters: Outlining Their Positive Applications. J. Vet. Behav..

[37] Powell L., Reinhard C., Satriale D., Morris M., Serpell J., Watson B. (2021). Characterizing Unsuccessful Animal Adoptions: Age and Breed Predict the Likelihood of Return, Reasons for Return and Post-Return Outcomes. Sci. Rep..

[38] Friend J., Bench C. (2020). Evaluating Factors Influencing Dog Post-Adoptive Return in a Canadian Animal Shelter. Anim. Welf..

[39] Dixit S.K., Patel P., Yadav S., Kumar Verma N. (2022). Vomiting in Dogs-A Review. Intas Polivet.

[40] Herron M.E., Lord L.K., Hill L.N., Reisner I.R. (2007). Effects of Preadoption Counseling for Owners on House-Training Success among Dogs Acquired from Shelters. J. Am. Vet. Med. Assoc..

[41] Wells D.L., Hepper P.G. (1999). Prevalence of Disease in Dogs Purchased from an Animal Rescue Shelter. Vet. Rec..

[42] Summers J.F., O’Neill D.G., Church D., Collins L., Sargan D., Brodbelt D.C. (2019). Health-Related Welfare Prioritisation of Canine Disorders Using Electronic Health Records in Primary Care Practice in the UK. BMC Vet. Res..

[43] Boyd C., Jarvis S., McGreevy P., Heath S., Church D., Brodbelt D., O’Neill D. (2018). Mortality Resulting from Undesirable Behaviours in Dogs Aged under Three Years Attending Primary-Care Veterinary Practices in England. Anim. Welf..

[44] Martínez Á.G., Santamarina Pernas G., Diéguez Casalta F.J., Suárez Rey M.L., De la Cruz Palomino L.F. (2011). Risk Factors Associated with Behavioral Problems in Dogs. J. Vet. Behav. Clin. Appl. Res..

[45] Bradshaw J.W.S., Goodwin D., Lea A.M., Whitehead S.L. (1996). A Survey of the Behavioural Characteristics of Pure-Bred Dogs in the United Kingdom. Vet. Rec..

[46] Bamberger M., Houpt K.A. (2006). Signalment Factors, Comorbidity, and Trends in Behavior Diagnoses in Cats: 736 Cases (1991–2001). J. Am. Vet. Med. Assoc..

[47] Mornement K.M., Coleman G.J., Toukhsati S.R., Bennett P.C. (2015). Evaluation of the Predictive Validity of the Behavioural Assessment for Re-Homing K9’s (B.A.R.K.) Protocol and Owner Satisfaction with Adopted Dogs. Appl. Anim. Behav. Sci..

[48] Scott S., Jong E., McArthur M., Hazel S.J. (2018). Follow-up Surveys of People Who Have Adopted Dogs and Cats from an Australian Shelter. Appl. Anim. Behav. Sci..

[49] Elliott R., Toribio J.-A.L.M.L., Wigney D. (2010). The Greyhound Adoption Program (GAP) in Australia and New Zealand: A Survey of Owners’ Experiences with Their Greyhounds One Month after Adoption. Appl. Anim. Behav. Sci..

[50] Döring D., Nick O., Bauer A., Küchenhoff H., Erhard M.H. (2017). How Do Rehomed Laboratory Beagles Behave in Everyday Situations? Results from an Observational Test and a Survey of New Owners. PLoS ONE.

[51] Appleby D.L., Bradshaw J.W.S., Casey R.A. (2002). Relationship between Aggressive and Avoidance Behaviour by Dogs and Their Experience in the First Six Months of Life. Vet. Rec..

