# Supplementary material for: New Strategies of Canine Post-Adoption Support: Methods for a Prospective Longitudinal Cohort Study
Source: Animals (Basel). 2025 Apr 27;15(9):1232. doi: 10.3390/ani15091232 (PMC12071166; doi:10.3390/ani15091232)
Supplement: Supplementary file 1 [file animals-15-01232-s001.zip › animals-3536526-supplementary.pdf]

Supplementary material

A full transcript of questions, responses and logic (action) for each call script at 2-day, 2-week and 4-month timepoints, respectively.

A. 2-day Call Script

Question Number	Text available for call centre agent	Response options	Action
Section 1: Availability, consent and dog updates			
1.1	Good morning/afternoon/evening, my name is XXX and I am calling from Dogs Trust. May I speak to [Owner's name] please? ***If registered owner not available, ask for another adult***	Yes, registered owner answered	Go to Q1.29
		No, registered owner not available, but consenting adult available	
		No, registered owner not available. No consenting adult available	End call
1.2	I understand that you very recently adopted a dog called [Dog's name] from [Rehoming centre]. Is [he/she] still with you?	Yes, owner still has the dog	Go to Q1.4
		No, owner no longer has dog	Go to Q1.3
1.3	Do you mind telling me what has happened to [him/her]?	Returned to centre	End call
		Passed to another private individual	
		Homed to a different rehoming charity	
		Euthanised for behaviour reasons	
		Euthanised due to road or other accident	
		Euthanised due to illness or disease	Text box for details; End call
		Other reason, please specify…..	
I should explain that I am calling from a central team at Dogs Trust rather than from [Rehoming centre], so sadly I haven't met [Dog's name] myself – the rehoming centre team are very busy caring for our dogs, and I'm helping out to check how things are going. As part of the adoption process you agreed for us to contact you by telephone to check on how [Dog's name] is.			
1.4	Could I ask if [Dog's name] is still [his/her] name or have you changed it?	No, the dog's name is the same	Go to Q1.5
		Yes, the dog's name has changed	Text box for new name; Go to Q1.5
1.5	Are you happy for me to ask some simple questions about how [Dog's name] is settling in with you now? It should take no more than 5-10 minutes.	Yes, they can answer questions now	Go to Section 2
		No, they would like a call at another time	End call
		No, they don't want a call at all	Go to Q1.6
		No, they are generally unhappy about contact or service and want no more contact	Text box for details; End call
		No, they are generally unhappy about contact and would like to talk to someone.	
1.6	That's absolutely fine, but before I finish could I just check that you have no concerns with [Dog's name] dog name that you would like our help with?<br>Is the problem related to [Dog's name]'s health or behaviour, or both?	No problems reported	Check email consent; End call
		Only health problems reported	Go to Section 2 ONLY
		Only behaviour problems reported	Go to Section 3 ONLY
		Health and behaviour problems reported	Go to Section 2 then Section 3
		No, maintain no consent	End call
Section 2: Dog health			
I'm going to start off with a few questions about [Dog's name] health. I am not myself a qualified veterinary practitioner, so cannot give you advice directly. However, based on your responses to these questions, I can ask a colleague from the veterinary team to call you back if necessary.			
2.1	Would you say [Dog's name] has been eating OK since you picked [him/her] up?	No, he/she has not eaten at all	Go to Q2.2
		No, he/she has only eaten very small amounts	
		Yes, he/she is frequently begging, stealing human food or raiding the bin	Go to 2.3
		No, he/she will only eat specially cooked food, not his/her normal dog food	Go to Q2.4
		No, he /she has been eating but is picky	
		Yes, he/she has been eating OK	
		Yes, he/she is eating well/very well	

		Yes, he/she seems ravenous	
		Other response (with Text box)	
2.2	Have you noticed any sickness, diarrhoea, listlessness or lethargy as well as [his/her] poor appetite?	Yes, he/she has vomited	24hr Vet call; Go to Q2.11
		Yes, he/she has had diarrhoea	
		Yes, he/she has had vomiting and diarrhoea	
		Yes, he/she has been lethargic or listless	
		Yes, he/she has been lethargic/listless and had vomiting/diarrhoea	
		No, none of these noticed	Go to Q2.11
		Other response (with Text box)	
2.3	Is [Dog's name] stealing or begging causing you problems, or something that you are struggling to cope with?	No, the behaviour is not serious/not a problem	Go to Q2.4
		Yes, the behaviour is a problem	72h Behaviour call; Go to Q2.4
2.4	Has [Dog's name] been sick or vomited since coming to live with you? (If 'Yes': Was this food or something else? And how often has it happened?)	Yes, he/she has brought up food once	Go to Q2.5
		Yes, he/she has brought up saliva/spit/mucous once	
		Yes, he/she has brought up something they ate once	
		Yes, he/she has brought up food one more than one occasion	24hr Vet call; Go to Q2.6
		Yes, he/she has brought up saliva/spit/mucous more than once	
		Yes, he/she has brought up something they ate more than once	
		No, no vomiting or sickness seen	Go to Q2.6
		Other response (with Text box)	
2.5	Have you noticed any diarrhoea, listlessness or lethargy as well as [his/her] sickness?	Yes, he/she has had diarrhoea	24hr Vet call; Go to Q2.11
		Yes, he/she has been lethargic or listless	
		Yes, he/she has been lethargic/listless and had vomiting/diarrhoea	
		No, none of these seen	Go to Q2.11
		Other response (with Text box)	
2.6	Has [Dog's name] had diarrhoea or sloppy stools/poo since arriving home? (If YES: I'm sorry to hear that. Is it very runny? And how often has it happened?)	Yes, stools/poo have been a bit loose on one or more occasion	Go to Q2.7
		Yes, he/she has had runny diarrhoea on one occasion only	
		Yes, he/she has had runny diarrhoea all the time since arriving home	24hr Vet call; Go to Q2.11
		Yes, he/she had runny diarrhoea when they first arrived but it has now improved	Go to Q2.8
		No, no diarrhoea or loose stools noticed	
		Other response (with Text box)	
2.7	Have you noticed any listlessness or lethargy as well as [his/her] the diarrhoea or loose stools?	No, he/she hasn't been lethargic or listless	Go to Q2.11
		Yes, he/she has been lethargic or listless	24hr Vet call; Go to Q2.11
		Other response (with Text box)	Text box; Go to Q2.11
2.8	Would you say that [Dog's name] has been particularly lethargic or listless since coming to live with you?	No, he/she 's been so lively/bouncy he's been difficult to handle	Go to Q2.9
		Yes, he/she seems worried about things and not wanting to do things	
		Yes, he/she has been a bit quiet	Go to Q2.10
		Yes, he/she has been very listless/lethargic	
		Yes, he/she has been unwilling to do anything	
		No, no problems	Go to Q2.11
		No, he/she 's been energetic/lively/bouncy	
		Other response (with Text box)	
2.9	Is [Dog's name] behaviour causing you problems, or something that you are struggling to cope with?	No, the behaviour is not serious/not a problem	Go to Q2.11
		Yes, the behaviour is a problem	72h Behaviour call; Go to Q2.11

2.10	Have you noticed any other signs as well as [his/her] general lethargy or listlessness?	No, no other signs	Go to Q2.11
		Yes, other signs of poor health	24hr Vet call; Go to Q2.11
		Yes, other behavioural signs	24h Behaviour call; Go to Q2.11
2.11	Has [Dog's name] been coughing or had difficulty breathing since coming to live with you?	Yes, he/she has coughed on one or two occasions, but no difficulty breathing	Go to Q2.12
		Yes, he/she seems to have had difficulty breathing on one or two occasions, but is not coughing	
		Yes, he/she has been coughing on more than a couple of occasions, but has no difficulty breathing	24hr Vet call; Go to Q2.13
		Yes, he/she has had difficulty breathing on more than a couple of occasions, but has no coughing	
		Yes, he/she has been coughing and has difficulty breathing	
		No, no coughing or difficulty breathing	Go to Q2.13
		Other response (with Text box)	
2.12	Could you tell me in what situation you noticed this happening?	During exercise, on a walk or during play	24hr Vet call; Go to Q2.13
		When he/she pulled on a collar or when I pulled back on the lead	
		During eating	Go to Q2.13
		When chewing up a toy or other non-food item	
		Other response (with Text box)	
2.13	Has [Dog's name] appeared to collapse, fallen over without reason, been unable to get up or unable to exercise since coming to live with you?	Yes, he/she has collapsed	24hr Vet call; Go to Q2.14
		Yes, he/she has fallen over without reason	
		Yes, he/she has seemed to be reluctant to, or be unable to, get up	
		Yes he/she has seemed to be unable to exercise	
		No, none of these have occurred	Go to Q2.14
		Other response (with Text box)	
2.14	Is there anything else about [Dog's name] health that you are worried about? Is this something you would like some help or advice with?	Yes, other concerns and would like advice	24hr Vet call; Go to section 3
		Yes, other concerns but don't want advice	Go to section 3
		No, no other concerns	
Section 3: Dog behaviour			
Next I'm going to ask a few questions about [Dog's name]'s behaviour during the last month. Again these questions will really help us to understand the how our dogs are settling in their new homes but Dogs Trust also provides lifelong behaviour advice for all of our dogs so we can arrange to get you some help if you would like. I'll be asking about a range of behaviours which you may or may not have seen – please bear with me as I run through them. I am not a qualified behaviourist, so cannot give you advice directly. However, based on your responses to these questions, I can ask one of my colleagues from our behaviour team to call you back if there are any issues we can help you with.			
3.1	Have you seen [Dog's name] doing any of the following behaviours since [he/she] was adopted? Growling, baring teeth or wrinkling lips up, snapping, biting or nipping, lunging forward whilst barking, grabbing (e.g. grabbing lead), mouthing (e.g. at sleeves or arms) or standing very still and staring?	No aggression seen	Go to Q3.6
		Yes, at least one of these signs have occurred	Go to Q3.2
3.2	Which of those behaviour(s) have you seen? Growling, baring teeth or wrinkling lips up, snapping, biting, nipping, lunging forward whilst barking, grabbing (e.g. grabbing lead), mouthing (e.g. at sleeves or arms) or standing very still and staring	Growling	24hr Behaviour call; Go to Q3.3
		Baring teeth	
		Wrinkling up lips	
		Snapping	
		Biting/Nipping	
		Lunging forward whilst barking	
		Grabbing (e.g. grabbing lead)	
		Mouthing (e.g. at sleeves or arms)	
		Standing very still and staring	
		Other behaviour (with Text box)	

		Text box for more information	
3.3	Could you tell me who or what [Dog's name] was reacting to when you noticed this behaviour?	Towards owner or an adult member of the household	24hr Behaviour call; Go to Q3.4
		Towards a child member of the household	
		Towards an adult visitor to the house	
		Towards a child visitor to the house	
		Towards an unfamiliar visitor to the house	
		Towards a non-household adult when out	
		Towards a non-household child when out	
		Towards another household dog	
		Towards an unfamiliar dog	
		Towards household cat	
		Towards other pets	72hr Behaviour call; Go to Q3.4
		Towards livestock or horses	
		Towards wildlife or local cats	
		Towards an inanimate object (e.g. lead)	
		In response to a noise or sound	
		In response to a flying insect (e.g. fly or bee)	
		Other target (with Text box)	
		Text box for more information	
3.4	To provide more information for my colleague, would you be able to tell me the situation in which you noticed this behaviour?	Without any apparent warning	Go to Q3.5
		Without any apparent reason	
		When dog was eating or around food	
		When dog was sleeping or resting	
		When dog was approached	
		When dog was reacting to another animal	
		When lead, collar or harness was put on	
		When dog was told off	
		When dog was put in another room/in an indoor kennel/behind a baby gate	
		When someone rang the doorbell or knocked at the door	
		When a person or owner came into the house	
		When being picked up or during close handling (e.g. drying feet, cuddling)	
		When member of household withdrew attention/stopped making a fuss	
		In response to a jogger or cyclist	
		When something was seen through a window or door	
		Don't know/can't remember	
		Other situation (with Text box)	
		Text box for more information	
3.5	I'm going to ask you next about how [Dog's name] reacts to being left alone. Firstly, can I ask have you left [Dog's name] at home without human company yet? If yes, was this with another dog?	Not applicable, haven't left him/her alone	Go to Q3.6a
		Yes, dog has been left alone but only with another dog	Go to Q3.6b
		Yes, dog has been left alone, without another dog	
		Yes, dog has been left alone, sometimes with and sometimes without another dog	
3.6a	Even though you haven't left [Dog's name] alone, have you noticed that [Dog's name] has shown any of the following behaviours when [he/she] was separated from you or a member of the family, for example when you entered a different room or into the garden or closed the door overnight or during the day? ***List out the behaviour signs in response options***	No signs noticed	Go to Q3.7
		Toileting (wee or poo) found on return	24hr Behaviour call; Go to Q3.8
		Owner heard dog barking as they were leaving	
		Owners heard howling on leaving	
		Chewing or scratching around doorway heard after owner left	
		Owners heard barking as they returned	
		Owners heard howling as they returned	
		Barking heard by neighbours whilst out	
		Howling heard by neighbours whilst out	
		Whining or whimpering	
		Panting when leaving	
		Vomiting or drooling	

		Toileting (weeing or pooing) as leaving	
		Owners found damage around doorway on return	
		Preventing owners from leaving (e.g. blocking doorway)	
		Pacing, spinning, circling or tail chasing	
		Items other than toys found chewed or destroyed on return	48hr Behaviour call; Go to Q3.8
		Trying to get out through door with owners	
		Excitability or excessive greeting when owner returned	
		Problems getting back into the house on return	
		Other behaviour(s) when left	24-48hr Behaviour call; Go to Q3.8
		Text box for more information	
3.6a	Have you noticed that [Dog's name] has shown any of the following behaviours when [he/she] was left alone? ***List out the behaviour signs in response options***	No signs noticed	Go to Q3.9
		Toileting (wee or poo) found on return	24hr Behaviour call; Go to Q3.9
		Owner heard dog barking as they were leaving	
		Owners heard howling on leaving	
		Chewing or scratching around doorway heard after owner left	
		Owners heard barking as they returned	
		Owners heard howling as they returned	
		Barking heard by neighbours whilst out	
		Howling heard by neighbours whilst out	
		Whining or whimpering	
		Panting when leaving	
		Vomiting or drooling	
		Toileting (weeing or pooing) as leaving	
		Owners found damage around doorway on return	
		Preventing owners from leaving (e.g. blocking doorway)	
		Pacing, spinning, circling or tail chasing	
		Items other than toys found chewed or destroyed on return	48hr Behaviour call; Go to Q3.8
		Trying to get out through door with owners	
		Excitability or excessive greeting when owner returned	
		Problems getting back into the house on return	
		Other behaviour(s) when left	24-48hr Behaviour call; Go to Q3.8
		Text box for more information	
3.7	Do you have any concerns about leaving your dog; would you like any advice on this?	Yes, some concerns, would like advice (with Text box)	48hr Behaviour call; Go to Q3.8
		Yes, some concerns but no advice needed	Go to Q3.8
		No concerns	
3.8	Have you noticed any other behaviours that you found a problem or were concerned about? If so, would you like any advice from our behaviour team?	Yes, other signs but don't need help	Go to Q3.9
		Yes, other signs and would like help	Go to Q3.11
		No, no other signs	Go to section 4
3.9	Would you mind telling me which behaviours you have seen – just so I can make a note ?	Signs of aggression (Growling, baring teeth or wrinkling lips up, snapping, biting, nipping, lunging forward whilst barking, grabbing (e.g. grabbing lead), mouthing (e.g. at sleeves or arms) or standing very still and staring)	Go to Q3.10
		Problems when left alone or when separated from owner/family member e.g. barking, howling, whining, toileting, destruction	
		Difficult to walk on the lead e.g. pulling on the lead or refuses to walk	
		Toileting inside the home	
		Barks excessively e.g. in the garden	

		Barks at people or animals	
		Chews or destroys non-food items other than own toys	
		Mounts/humps person, dog, object (e.g. bed)	
		Follows people around house and/or trips people up	
		Won't get into or out of the car	
		Won't get off sofa/other furniture	
		Gets on furniture and we don't want them to	
		Steals food, scavenges or raids bins	
		Steals items other than own toys	
		Not giving up items including toys	
		Doesn't come back when called	
		Reacts to noises	
		Over active/doesn't settle down	
		Jumping up	
		Bothers another household dog	
		Bothers a non-household dog	
		Chases other dogs	
		Chases household cats or other pets	
		Chases local cats or wildlife	
		Chases joggers	
		Chases bikes	
		Chases vehicles	
		Chases livestock or horses	
		Restless or vocalising in car	
		Cowers, hides or runs away from moving vehicles	
		Digging	
		Toileting issue outside of the home	
		Is sick or drools during travelling	
		Escaping	
		Spins in circles or chases tail repetitively	
		Paces a lot	
		Barks at inanimate object e.g. cars/reflection	
		Plays excessively/roughly with another dog	
		Uninterested in play/reluctant to play	
		Wants to play excessively e.g. with toys	
		Pawing at or licking people	
		Shies away from people e.g. runs away/hides	
		Runs away/hides from other dogs	
		Seems fixated on or chases points of light or shadows	
		Stares at walls or 'nothing'	
		Seems unreactive	
		Stares at people	
		Won't go up or down stairs	
		Other behaviours (with Text box)	
		Text box for more information	
		Rather not say	Go to section 4
3.10	Are you sure you don't need any help from us for that? I'm happy to ask a colleague to give you a call if it would be useful?	No, don't need help	Go to section 4
		Yes, please arrange call	
3.11	That's absolutely fine. Could you let me know what problem you're having, and I can arrange a call back for you	Signs of aggression (Growling, baring teeth or wrinkling lips up, snapping, biting, nipping, lunging forward whilst barking, grabbing (e.g. grabbing lead), mouthing (e.g. at sleeves or arms) or standing very still and staring)	24hr Behaviour call; Go to Section 4
		Problems when left alone or when separated from owner/family member e.g. barking, howling, whining, toileting, destruction	
		Not giving up items including toys	

		Bothers another household dog	
		Seems fixated on or chased points of light or shadows	
		Stares at walls or 'nothing'	
		Spins in circles or chases tail repetitively	
		Chases household cats or other pets	
		Toileting inside the home	48hr Behaviour call; Go to Section 4
		Barks excessively e.g. in the garden	
		Won't get off sofa/other furniture	
		Reacts to noises	
		Over active/doesn't settle down	
		Jumping up	
		Bothers a non-household dog	
		Chases other dogs	
		Chases joggers	
		Chases bikes	
		Chases livestock or horses	
		Cowers, hides or runs away from moving vehicles	
		Escaping	
		Plays excessively/roughly with another dog	
		Shies away from people e.g. runs away/hides	
		Mounts/humps person, dog, object (e.g. bed)	
		Difficult to walk on the lead e.g. pulling on the lead or refuses to walk	72hr Behaviour call; Go to Section 4
		Barks at people or animals	
		Chews or destroys non-food items other than own toys	
		Follows people around house and/or trips people up	
		Won't get into or out of the car	
		Gets on furniture and we don't want them to	
		Steals food, scavenges or raids bins	
		Steals items other than own toys	
		Doesn't come back when called	
		Chases local cats or wildlife	
		Chases vehicles	
		Restless or vocalising in car	
		Digging	
		Toileting issue outside of the home	
		Is sick or drools during travelling	
		Paces a lot	
		Barks at inanimate object e.g. cars/reflection	
		Uninterested in play/reluctant to play	
		Wants to play excessively e.g. with toys	
		Pawing at or licking people	
		Runs away/hides from other dogs	
		Seems unreactive	
		Stares at people	
		Won't go up or down stairs	
		Rather not say	
		Other behaviours (with Text box)	
		Text box for more information	
Section 4: Adoption Experience			
Finally, I'm going to ask you a few questions about your experiences when adopting [Dog's name].			
4.1	Would you say overall, you're happy that you decided to adopt [Dog's name]?	Not sure, depends on his/her behaviour	Go to Q4.2
		Not sure, too early to say	
		Not sure, it depends on his/her health	
		Not sure, it depends on his/her behaviour and health	
		No, unhappy about adoption for non-behaviour/health reasons	Go to Q4.3
		Yes, happy to have adopted dog	Go to Q4.4
		Other response (with Text box)	

4.2	Have we already discussed any behaviour or health concerns that are worrying you in this call? If no: Would you like some help with your concerns about [Dog's name] health or behaviour?	Yes, already discussed	Go to Q4.4
		No, owner has behaviour/health concerns not discussed but doesn't want advice	
		No, owner has health concerns not discussed and does want advice	24hr Vet call; Go to Q4.4
		No, owner has behaviour concerns not discussed and does want advice	48hr Behaviour call; Go to Q4.4
		No, owner has behaviour/health concerns not discussed and does want advice (with Text box)	24-48hr Vet and/or Behaviour call; Go to Q4.4
4.3	Would you mind telling me why you're not happy about adopting [Dog's name]?	Unhappy about the process	Text box for details; Go to Q4.4
		Wanted a different dog	
		Unhappy about customer service	
		Unexpected challenges of having a dog	
		Impact on lifestyle	
		Family member allergic to pet	
		Dog not getting on with other pets	
		Other response (with Text box)	
4.4	Overall, would you say your experience at the [Rehoming Centre] was very positive, quite positive, neutral, quite negative or very negative?	Very positive	Text box for details; Go to Q4.5
		Quite positive	
		Neutral	
		Quite negative	
		Very negative	
4.5	Thank you so much for speaking with me today, it is amazing to hear how [Dog's name] is getting on. We really appreciate your time, and I hope the call has been useful for you. We'll be back in touch in a couple of weeks. Please do call us back if you have any more worries in the meanwhile. Goodbye.		End Call

B. 2-week Call Script

Question Number	Text available for call centre agent	Response options	Action
Section 1: Availability, consent and dog updates			
1.1	Good morning/afternoon/evening, my name is XXX and I am calling from Dogs Trust. May I speak to [Owner's name] please? ***If registered owner not available, ask for another adult***	Yes, registered owner answered	Go to Q1.29
		No, registered owner not available, but consenting adult available	
		No, registered owner not available. No consenting adult available	End call
1.2	I am calling to check how [Dog's name] is getting on. Is [Dog's name] still with you?	Yes, owner still has the dog	Go to Q1.4
		No, owner no longer has dog	Go to Q1.3
1.3	Do you mind telling me what has happened to [him/her]?	Returned to centre	End call
		Passed to another private individual	
		Homed to a different rehoming charity	
		Euthanised for behaviour reasons	
		Euthanised due to road or other accident	
		Euthanised due to illness or disease	Text box for details; End call
		Other reason, please specify…..	
Thanks for chatting to us about [Dog's name]. I'm calling to check that things are going OK. I am calling from a central team at Dogs Trust rather than from so sadly I haven't met [Dog's name] myself – the rehoming centre team are very busy caring for our dogs, and I'm helping out to check how things are going.			
1.4	Can I check whether you have changed the name of the dog you adopted from us?	No, the dog's name is the same	Go to Q1.5
		Yes, the dog's name has changed	Text box for new name; Go to Q1.5
1.5	Are you happy for me to ask some simple questions about how [Dog's name] is settling in with you now? It should take no more than 5-10 minutes.	Yes, they can answer questions now	*If receiving ongoing support:* Go to 1.6; *If not receiving ongoing support:* Go to Section 2
		No, they don't want a call at all	End call
		No, they are generally unhappy about contact or service and want no more contact	
		No, they would like a call at another time	
		No, they are generally unhappy about contact and would like to talk to someone about this	Text box for details; End call
1.6	Are you happy with the support that you have been getting or can I ask someone to get back in touch with you for further help?	Happy with support, but would like further behaviour advice	Text box for details; 24hr Behaviour call; End call
		Happy with support, but would like more vet advise	Text box for details; End call
		Happy with support but need more behaviour and vet advice	
		Happy with support and don't need further help	
		Not happy with advise and need more behaviour advice	
		Not happy with advice and need more vet advice	
		Not happy with advice and need more vet and behaviour advice	
		Not happy with advice but don't need further advice	
		Don't know/Unsure	
Section 2: Dog health			
I'm going to ask a few questions about [Dog's name]'s health during the last month. I am not qualified to give veterinary advice, and I would advise you to speak to your vet about any health worries with [Dog's name]. The questions in this section will really help us to understand more about the health of Dogs Trust dogs as they settle into their new homes.			
2.1	Excluding the first few days after adoption, has [Dog's name] been unwell at all since you adopted?	Yes, do has had health problems	Go to Q2.2
		No health problems reported	Go to Q2.4
2.2	Would you mind telling me what health problems [Dog's name] has had?	Lethargic/listless	Text box for details; Go to Q2.3
		Off food	
		Vomiting	

		Diarrhoea	
		Coughing or difficulty breathing	
		Choking	
		Collapsing or not tolerating exercise	
		Dog collapsed, fallen over, unable to get up, unable to exercise since adoption	
		Other problem, please specify	
2.4	Have you taken [Dog's name] to your vet for this problem, and has [he/she] recovered?	No, not been to vet and dog still unwell	Text box for details; Go to Q2.4
		No, not been to vet yet but owner intends to	
		No, not been to vet but dog has recovered	
		Yes, been to vet and dog recovered	
		Yes, been to the vet and treatment ongoing	
		Other response, please specify	
2.5	Have you registered [Dog's name] with your local vet yet?	No, not registered with vet	Go to Section 3
		Yes, registered with vet	
		Not yet registered but planning to	
Section 3: Dog behaviour			
Next I'm going to ask a few questions about [Dog name]'s behaviour during the last month. Again these questions will really help us to understand the how our dogs are settling in their new homes but Dogs Trust also provides lifelong behaviour advice for all of our dogs so we can arrange to get you some help if you would like. I'll be asking about a range of behaviours which you may or may not have seen – please bear with me as I run through them. I am not a qualified behaviourist, so cannot give you advice directly. However, based on your responses to these questions, I can ask one of my colleagues from our behaviour team to call you back if there are any issues we can help you with.			
3.1	Have you seen [Dog's name] doing any of the following behaviours since [he/she] was adopted? Growling, baring teeth or wrinkling lips up, snapping, biting or nipping, lunging forward whilst barking, grabbing (e.g. grabbing lead), mouthing (e.g. at sleeves or arms) or standing very still and staring?	No aggression seen	Go to Q3.5
		Yes, at least one of these signs have occurred	Go to Q3.2
3.2	Which of those behaviour(s) have you seen? Growling, baring teeth or wrinkling lips up, snapping, biting, nipping, lunging forward whilst barking, grabbing (e.g. grabbing lead), mouthing (e.g. at sleeves or arms) or standing very still and staring	Growling	24hr Behaviour call; Go to Q3.3
		Baring teeth	
		Wrinkling up lips	
		Snapping	
		Biting/Nipping	
		Lunging forward whilst barking	
		Grabbing (e.g. grabbing lead)	
		Mouthing (e.g. at sleeves or arms)	
		Standing very still and staring	
		Other behaviour (with Text box)	
		Text box for more information	
3.3	Could you tell me who or what [Dog's name] was reacting to when you noticed this behaviour?	Towards owner or an adult member of the household	24hr Behaviour call; Go to Q3.4
		Towards a child member of the household	
		Towards an adult visitor to the house	
		Towards a child visitor to the house	
		Towards an unfamiliar visitor to the house	
		Towards a non-household adult when out	
		Towards a non-household child when out	
		Towards another household dog	
		Towards an unfamiliar dog	
		Towards household cat	
		Towards other pets	72hr Behaviour call; Go to Q3.4
		Towards livestock or horses	
		Towards wildlife or local cats	
		Towards an inanimate object (e.g. lead)	
		In response to a noise or sound	
		In response to a flying insect (e.g. fly/bee)	
		Other target (with Text box)	
		Text box for more information	
3.4	To provide more information for my colleague, would you be able to tell me	Without any apparent warning	Go to Q3.5
		Without any apparent reason	
		When dog was eating or around food	

	the situation in which you noticed this behaviour?	When dog was sleeping or resting	
		When dog was approached	
		When dog was reacting to another animal	
		When lead, collar or harness was put on	
		When dog was told off	
		When dog was put in another room/in an indoor kennel/behind a baby gate	
		When someone rang the doorbell or knocked at the door	
		When a person or owner came into the house	
		When being picked up or during close handling (e.g. drying feet, cuddling)	
		When member of household withdrew attention/stopped making a fuss	
		In response to a jogger or cyclist	
		When something was seen through a window or door	
		Don't know/can't remember	
		Other situation (with Text box)	
		Text box for more information	
3.5	I'm going to ask you next about how [Dog's name] reacts to being left alone. Firstly, can I ask have you left [Dog's name] at home without human company yet? If yes, was this with another dog?	Not applicable, haven't left him/her alone	Go to Q3.6a
		Yes, dog has been left alone but only with another dog	Go to Q3.6b
		Yes, dog has been left alone, without another dog	
		Yes, dog has been left alone, sometimes with and sometimes without another dog	
3.6a	Even though you haven't left [Dog's name] alone, have you noticed that [Dog's name] has shown any of the following behaviours when [he/she] was separated from you or a member of the family, for example when you entered a different room or into the garden or closed the door overnight or during the day? **List out the behaviour signs in response options**	No signs noticed	Go to Q3.7
		Toileting (wee or poo) found on return	24hr Behaviour call; Go to Q3.8
		Owner heard dog barking as they were leaving	
		Owners heard howling on leaving	
		Chewing or scratching around doorway heard after owner left	
		Owners heard barking as they returned	
		Owners heard howling as they returned	
		Barking heard by neighbours whilst out	
		Howling heard by neighbours whilst out	
		Whining or whimpering	
		Panting when leaving	
		Vomiting or drooling	
		Toileting (weeing or pooing) as leaving	
		Owners found damage around doorway on return	
		Preventing owners from leaving (e.g. blocking doorway)	
		Pacing, spinning, circling or tail chasing	
		Items other than toys found chewed or destroyed on return	48hr Behaviour call; Go to Q3.8
		Trying to get out through door with owners	
		Excitability or excessive greeting when owner returned	
		Problems getting back into the house on return	
		Other behaviour(s) when left	24-48hr Behaviour call; Go to Q3.8
		Text box for more information	
3.6a	Have you noticed that [Dog's name] has shown any of the following behaviours when [he/she] was left alone? **List out the behaviour signs in response options**	No signs noticed	Go to Q3.9
		Toileting (wee or poo) found on return	24hr Behaviour call; Go to Q3.9
		Owner heard dog barking as they were leaving	
		Owners heard howling on leaving	
		Chewing or scratching around doorway heard after owner left	

		Owners heard barking as they returned	
		Owners heard howling as they returned	
		Barking heard by neighbours whilst out	
		Howling heard by neighbours whilst out	
		Whining or whimpering	
		Panting when leaving	
		Vomiting or drooling	
		Toileting (weeing or pooing) as leaving	
		Owners found damage around doorway on return	
		Preventing owners from leaving (e.g. blocking doorway)	
		Pacing, spinning, circling or tail chasing	
		Items other than toys found chewed or destroyed on return	48hr Behaviour call; Go to Q3.8
		Trying to get out through door with owners	
		Excitability or excessive greeting when owner returned	
		Problems getting back into the house on return	
		Other behaviour(s) when left	24-48hr Behaviour call; Go to Q3.8
		Text box for more information	
3.7	Do you have any concerns about leaving your dog; would you like any advice on this?	Yes, some concerns, would like advice (with Text box)	48hr Behaviour call; Go to Q3.8
		Yes, some concerns but no advice needed	Go to Q3.8
		No concerns	
3.8	Have you noticed any other behaviours that you found a problem or were concerned about? If so, would you like any advice from our behaviour team?	Yes, other signs but don't need help	Go to Q3.9
		Yes, other signs and would like help	Go to Q3.11
		No, no other signs	Go to section 4
3.9	Would you mind telling me which behaviours you have seen – just so I can make a note ?	Signs of aggression (Growling, baring teeth or wrinkling lips up, snapping, biting, nipping, lunging forward whilst barking, grabbing (e.g. grabbing lead), mouthing (e.g. at sleeves or arms) or standing very still and staring)	Go to Q3.10
		Problems when left alone or when separated from owner/family member e.g. barking, howling, whining, toileting, destruction	
		Difficult to walk on the lead e.g. pulling on the lead or refuses to walk	
		Toileting inside the home	
		Barks excessively e.g. in the garden	
		Barks at people or animals	
		Chews or destroys non-food items other than own toys	
		Mounts/humps person, dog, object (e.g. bed)	
		Follows people around house and/or trips people up	
		Won't get into or out of the car	
		Won't get off sofa/other furniture	
		Gets on furniture and we don't want them to	
		Steals food, scavenges or raids bins	
		Steals items other than own toys	
		Not giving up items including toys	
		Doesn't come back when called	
		Reacts to noises	
		Over active/doesn't settle down	
		Jumping up	
		Bothers another household dog	
		Bothers a non-household dog	
		Chases other dogs	
		Chases household cats or other pets	
		Chases local cats or wildlife	

		Chases joggers	
		Chases bikes	
		Chases vehicles	
		Chases livestock or horses	
		Restless or vocalising in car	
		Cowers, hides or runs away from moving vehicles	
		Digging	
		Toileting issue outside of the home	
		Is sick or drools during travelling	
		Escaping	
		Spins in circles or chases tail repetitively	
		Paces a lot	
		Barks at inanimate object e.g. cars/reflection	
		Plays excessively/roughly with another dog	
		Uninterested in play/reluctant to play	
		Wants to play excessively e.g. with toys	
		Pawing at or licking people	
		Shies away from people e.g.runs away/hides	
		Runs away/hides from other dogs	
		Seems fixated on or chases points of light or shadows	
		Stares at walls or 'nothing'	
		Seems unreactive	
		Stares at people	
		Won't go up or down stairs	
		Other behaviours (with Text box)	
		Text box for more information	
		Rather not say	Go to section 4
3.10	Are you sure you don't need any help from us for that? I'm happy to ask a colleague to give you a call if it would be useful?	No, don't need help	Go to section 4
		Yes, please arrange call	
3.11	That's absolutely fine. Could you let me know what problem you're having, and I can arrange a call back for you	Signs of aggression (Growling, baring teeth or wrinkling lips up, snapping, biting, nipping, lunging forward whilst barking, grabbing (e.g. grabbing lead), mouthing (e.g. at sleeves or arms) or standing very still and staring)	24hr Behaviour call; Go to Section 4
		Problems when left alone or when separated from owner/family member e.g. barking, howling, whining, toileting, destruction	
		Not giving up items including toys	
		Bothers another household dog	
		Seems fixated on or chased points of light or shadows	
		Stares at walls or 'nothing'	
		Spins in circles or chases tail repetitively	
		Chases household cats or other pets	
		Toileting inside the home	48hr Behaviour call; Go to Section 4
		Barks excessively e.g. in the garden	
		Won't get off sofa/other furniture	
		Reacts to noises	
		Over active/doesn't settle down	
		Jumping up	
		Bothers a non-household dog	
		Chases other dogs	
		Chases joggers	
		Chases bikes	
		Chases livestock or horses	
		Cowers, hides or runs away from moving vehicles	
		Escaping	
		Plays excessively/roughly with another dog	

		Shies away from people e.g.runs away/hides	
		Mounts/humps person, dog, object (e.g. bed)	
		Difficult to walk on the lead e.g. pulling on the lead or refuses to walk	72hr Behaviour call; Go to Section 4
		Barks at people or animals	
		Chews or destroys non-food items other than own toys	
		Follows people around house and/or trips people up	
		Won't get into or out of the car	
		Gets on furniture and we don't want them to	
		Steals food, scavenges or raids bins	
		Steals items other than own toys	
		Doesn't come back when called	
		Chases local cats or wildlife	
		Chases vehicles	
		Restless or vocalising in car	
		Digging	
		Toileting issue outside of the home	
		Is sick or drools during travelling	
		Paces a lot	
		Barks at inanimate object e.g. car/reflections	
		Uninterested in play/reluctant to play	
		Wants to play excessively e.g. with toys	
		Pawing at or licking people	
		Runs away/hides from other dogs	
		Seems unreactive	
		Stares at people	
		Won’t go up or down stairs	
		Rather not say	
		Other behaviours (with Text box)	
		Text box for more information	
Section 4: Adoption Experience			
Finally, I’m going to ask you a few questions about your experiences when adopting [Dog’s name].			
4.1	Were you recommended to attend Dogs Trust Dog School when you adopted [Dog’s name]?	Yes, Dog School was recommended but I didn't attend	Go to Q4.2
		Yes, Dog School was recommended and I attended	Go to Q4.3
		No, Dog School was not recommended	Go to Q4.5
		Not sure/can't remember	
		Yes, Dog School was recommended and I plan to attend	
4.2	Could I ask the main reason why you decided not to attend?	Nearest class too far away	Go to Q4.5
		Classes were too expensive	
		I am an experienced trainer or prefer to train myself	
		I've been too busy	
		I've been to another trainer	
		Other, please specify (with Text box)	
4.3	Overall how would you rate your experience of Dog School?	Very positive	Go to Q4.4
		Quite positive	
		Neutral	
		Quite negative	
		Very negative	
4.4	Would you mind telling me what factors mainly influenced your rating of Dog School?	Text box for details	Go to Q4.5
4.5	Would you say overall, you’re happy that you decided to adopt [Dog’s name]?	Not sure, depends on his/her behaviour	Go to Q4.6
		Not sure, it depends on his/her health	
		Not sure, it depends on his/her behaviour and health	
		Not sure, too early to say	
		No, unhappy about adoption for non-behaviour/health reasons	Go to Q4.7

		Yes, happy to have adopted dog	Go to Q4.8
		Other response (with Text box)	
4.6	Have we already discussed any concerns that are worrying you in this call? If no, would you like some help?	Yes, already discussed	Go to Q4.8
		No, owner has behaviour/health concerns not discussed but doesn't want advice	
		No, owner has health concerns not discussed and does want advice	24hr Vet call; Text box for details; Go to Q4.8
		No, owner has behaviour concerns not discussed and does want advice	48hr Behaviour call; Text box for details; Go to Q4.8
		No, owner has behaviour/health concerns not discussed and does want advice	48hr Behaviour & Vet call; Text box for details; Go to Q4.8
4.7	Would you mind telling me why you're not happy about adopting [Dog's name]?	Unhappy about the process	Text box for details; Go to Q4.8
		Wanted a different dog	
		Unhappy about customer service	
		Unexpected challenges of having a dog	
		Other response	
		Impact on lifestyle	
		Family member allergic to pet	
		Dog not getting on with other pets	
4.8	Thank you so much for speaking with me today, it is amazing to hear how [Dog's name] is getting on. We really appreciate your time, and I hope the call has been useful for you. We'll be back in touch in a couple of weeks. Please do call us back if you have any more worries in the meanwhile. Goodbye.		End Call

C. 4-week Call Script

Question Number	Text available for call centre agent	Response options	Action
Section 1: Availability, consent and dog updates			
1.1	Good morning/afternoon/evening, my name is XXX and I am calling from Dogs Trust. May I speak to [Owner's name] please? ***If registered owner not available, ask for another adult***	Yes, registered owner answered	Go to Q1.29
		No, registered owner not available, but consenting adult available	
		No, registered owner not available. No consenting adult available	End call
1.2	I understand you adopted a dog from [Rehoming Centre] around 4 months ago. Is [Dog's name] still with you?	Yes, owner still has the dog	Go to Q1.4
		No, owner no longer has dog	Go to Q1.3
1.3	Do you mind telling me what has happened to [him/her]?	Returned to centre	End call
		Passed to another private individual	
		Homed to a different rehoming charity	
		Euthanised for behaviour reasons	
		Euthanised due to road or other accident	
		Euthanised due to illness or disease	Text box for details; End call
		Other reason, please specify…..	
I'm calling to check that things are going OK for you and to see if there is anything we can help you with. I am calling from a central team at Dogs Trust rather than from [Rehoming Centre] so sadly I haven't met [Dog's name] myself but I'm helping out to check how things are going with our adopted dogs.			
1.4	Can I check whether you have changed the name of the dog you adopted from us?	No, the dog's name is the same	Go to Q1.5
		Yes, the dog's name has changed	Text box for new name; Go to Q1.5
1.5	Are you happy for me to ask some simple questions about how [Dog's name] is settling in with you now? It should take no more than 5-10 minutes.	Yes, they can answer questions now	*If receiving ongoing support:* Go to 1.6; *If not:* Go to Section 2
		No, they don't want a call at all	End call
		No, they are generally unhappy about contact or service and want no more contact	
		No, they would like a call at another time	
		No, they are generally unhappy about contact and would like to talk to someone about this	Text box for details; End call
1.6	*** Only if owner is receiving ongoing behaviour support **<br><br>Are you happy with the support that you have been getting or can I ask someone to get back in touch with you for further help?	Happy with support, but would like further behaviour advice	48hr Behaviour call; Text box for details; Go to 1.7
		Happy with support and don't need further help	
		Not happy with advise and need more behaviour advice	
		Don't know/Unsure	
		Not happy with advice but don't need further advice	48hr Behaviour call; Text box for details; Go to Section 2
1.7	Apart from the issue that you have recently spoken about with our behaviour team, has [Dog's name] shown any other behaviours that you find a problem or are concerned about? If so would you like any further advice from our behaviour team?	Yes, other signs and would like help	48hr Behaviour call; Text box for details; Go to Section 2
		No, no other signs	Text box for details; Go to Section 2
		Yes, other signs but don't need help	
Section 2: Dog health			
I'm going to ask a few questions about [Dog's name]'s health during the last month. I am not qualified to give veterinary advice, and I would advise you to speak to your vet about any health worries with [Dog's name]. The questions in this Section will really help us to understand more about the health of Dogs Trust dogs as they settle into their new homes.			
2.1	In the last month, have you noticed any problems with [Dog's name]'s skin or coat at all? For example, any itchiness, scabs, broken skin, dandruff or a dull, dry or greasy coat?	Itchiness	Go to Q2.2
		Scabs	
		Broken skin	
		Dandruff	
		Dull coat	
		Dry coat	
		Greasy coat	
		Other, please specify (with Text box)	
		No problems seen in last month	Go to Q2.3

		Not sure/don't know	
2.2	Has this problem been treated at all, and is this still ongoing?	Yes - treated and no longer a problem	Go to Q2.3
		Yes - treated and has improved	
		Yes - treated but not improved	
		Yes - treated and not sure if it has improved	
		No - not treated but no longer a problem	
		No - not treated but it has improved	
		No - not treated and has not improved	
		No - not treated and not sure if it has improved	
		Not sure/don't know	
		Text box for more information	
2.3	In the last month, how often - if at all - would you say that [Dog's name] has suffered from any vomiting or diarrhoea?	Vomiting only - on one or two occasions	Go to Q2.4
		Diarrhoea only - on one or two occasions	
		Vomiting and diarrhoea on one or two occasions	
		Vomiting only - on three or more occasions	
		Diarrhoea only - on three or more occasions	
		Vomiting and diarrhoea on three or more occasions	
		Other, please specify (with Text box)	
		No vomiting or diarrhoea seen	Go to Q2.5
		Not sure/don't know	
2.4	Has this been treated at all, and is this still ongoing?	Yes - treated and no longer a problem	Go to Q2.5
		Yes - treated and has improved	
		Yes - treated but not improved	
		Yes - treated and not sure if it has improved	
		No - not treated but no longer a problem	
		No - not treated but it has improved	
		No - not treated and has not improved	
		No - not treated and not sure if it has improved	
		Other, please specify (with Text box)	
2.5	In the last month has [Dog's name] had any other health problems? For example, coughing or difficultly breathing, collapsing, any problems with ears, eyes or mouth or any limping/lameness	Yes, new health problems in the last month	Go to Q 2.6
		Health problems reported previously but no new problems within the last month	Go to Q 2.8
		No other health problems in the last month	
		Not sure/don't know	
2.6	Would you mind telling me what health problems [he/she] has had?	Lethargic / listless	Go to Q2.7
		Off food	
		Coughing or difficulty breathing	
		Choking	
		Dog collapsed, fallen over, unable to get up, unable to exercise	
		Small cut or injury	
		Eye problem e.g. conjunctivitis	
		Ear problem e.g. infection	
		Coughing	
		Sneezing	
		Mouth/teeth problems	
		Lameness/limb problem	
		Other response, please specify (with Text box)	
		Text box for more information	
2.7	Have you taken [Dog's name] to your vet for this problem, and has [he/she] recovered?	No, not been to vet and dog still unwell	Go to Q 2.8
		No, not been to vet yet but owner intends to	
		No, not been to vet but dog has recovered	
		Yes, been to vet and dog recovered	
		Yes, been to the vet and issue ongoing	
		Other response, please specify (with Text box)	
2.8	Have you given [Dog's name] any flea or worming treatment since you have adopted [him/her]?	Yes, only worming treatment	Go to Section 3
		Yes, only flea treatment	
		Yes both worming and flea treatment	
		No but plan to	
		No	
Section 3: Dog behaviour			

<table>
<tr><td colspan="4">Next I’m going to ask a few questions about [Dog name]'s behaviour during the last month. Again these questions will really help us to understand the how our dogs are settling in their new homes but Dogs Trust also provides lifelong behaviour advice for all of our dogs so we can arrange to get you some help if you would like. I’ll be asking about a range of behaviours which you may or may not have seen – please bear with me as I run through them. I am not a qualified behaviourist, so cannot give you advice directly. However, based on your responses to these questions, I can ask one of my colleagues from our behaviour team to call you back if there are any issues we can help you with.</td></tr>
<tr><td rowspan="2">3.1</td><td rowspan="2">Have you seen [Dog’s name] doing any of the following behaviours in the last month? Growling, baring teeth or wrinkling lips up, snapping, biting or nipping, lunging forward whilst barking, grabbing (e.g. grabbing lead), mouthing (e.g. at sleeves or arms) or standing very still and staring?</td><td>No aggression seen</td><td>Go to Q3.5</td></tr>
<tr><td>Yes, at least one of these signs have occurred</td><td>Go to Q3.2</td></tr>
<tr><td rowspan="11">3.2</td><td rowspan="11">Which of those behaviour(s) have you seen? Growling, baring teeth or wrinkling lips up, snapping, biting, nipping, lunging forward whilst barking, grabbing (e.g. grabbing lead), mouthing (e.g. at sleeves or arms) or standing very still and staring</td><td>Growling</td><td rowspan="11">24hr Behaviour call;
Go to Q3.3</td></tr>
<tr><td>Baring teeth</td></tr>
<tr><td>Wrinkling up lips</td></tr>
<tr><td>Snapping</td></tr>
<tr><td>Biting/Nipping</td></tr>
<tr><td>Lunging forward whilst barking</td></tr>
<tr><td>Grabbing (e.g. grabbing lead)</td></tr>
<tr><td>Mouthing (e.g. at sleeves or arms)</td></tr>
<tr><td>Standing very still and staring</td></tr>
<tr><td>Other behaviour (with Text box)</td></tr>
<tr><td>Text box for more information</td></tr>
<tr><td rowspan="18">3.3</td><td rowspan="18">Could you tell me who or what [Dog’s name] was reacting to when you noticed this behaviour?</td><td>Towards owner or an adult member of the household</td><td rowspan="10">24hr Behaviour call;
Go to Q3.4</td></tr>
<tr><td>Towards a child member of the household</td></tr>
<tr><td>Towards an adult visitor to the house</td></tr>
<tr><td>Towards a child visitor to the house</td></tr>
<tr><td>Towards an unfamiliar visitor to the house</td></tr>
<tr><td>Towards a non-household adult when out</td></tr>
<tr><td>Towards a non-household child when out</td></tr>
<tr><td>Towards another household dog</td></tr>
<tr><td>Towards an unfamiliar dog</td></tr>
<tr><td>Towards household cat</td></tr>
<tr><td>Towards other pets</td><td rowspan="8">72hr Behaviour call;
Go to Q3.4</td></tr>
<tr><td>Towards livestock or horses</td></tr>
<tr><td>Towards wildlife or local cats</td></tr>
<tr><td>Towards an inanimate object (e.g. lead)</td></tr>
<tr><td>In response to a noise or sound</td></tr>
<tr><td>In response to a flying insect (e.g. fly/bee)</td></tr>
<tr><td>Other target (with Text box)</td></tr>
<tr><td>Text box for more information</td></tr>
<tr><td rowspan="15">3.4</td><td rowspan="15">To provide more information for my colleague, would you be able to tell me the situation in which you noticed this behaviour?</td><td>Without any apparent warning</td><td rowspan="15">Go to Q3.5</td></tr>
<tr><td>Without any apparent reason</td></tr>
<tr><td>When dog was eating or around food</td></tr>
<tr><td>When dog was sleeping or resting</td></tr>
<tr><td>When dog was approached</td></tr>
<tr><td>When dog was reacting to another animal</td></tr>
<tr><td>When lead, collar or harness was put on</td></tr>
<tr><td>When dog was told off</td></tr>
<tr><td>When dog was put in another room/in an indoor kennel/behind a baby gate</td></tr>
<tr><td>When someone rang the doorbell or knocked at the door</td></tr>
<tr><td>When a person or owner came into the house</td></tr>
<tr><td>When being picked up or during close handling (e.g. drying feet, cuddling)</td></tr>
<tr><td>When member of household withdrew attention/stopped making a fuss</td></tr>
<tr><td>In response to a jogger or cyclist</td></tr>
<tr><td>When something was seen through a window or door</td></tr>
</table>

		Don't know/can't remember	
		Other situation (with Text box)	
		Text box for more information	
3.5	I'm going to ask you next about how [Dog's name] reacts to being left alone. Firstly, can I ask have you left [Dog's name] at home without human company in the last month? If yes, was this with another dog?	Not applicable, haven't left him/her alone	Go to Q3.6a
		Yes, dog has been left alone but only with another dog	Go to Q3.6b
		Yes, dog has been left alone, without another dog	
		Yes, dog has been left alone, sometimes with and sometimes without another dog	
		Skip/prefer not to say	Go to Q3.8
3.6a	Even though you haven't left [Dog's name] alone, have you noticed that [Dog's name] has shown any of the following behaviours when [he/she] was separated from you or a member of the family, for example when you entered a different room or into the garden or closed the door overnight or during the day? ***List out the behaviour signs in response options***	No signs noticed	Go to Q3.7
		Toileting (wee or poo) found on return	24hr Behaviour call; Go to Q3.8
		Owner heard dog barking as they were leaving	
		Owners heard howling on leaving	
		Chewing or scratching around doorway heard after owner left	
		Owners heard barking as they returned	
		Owners heard howling as they returned	
		Barking heard by neighbours whilst out	
		Howling heard by neighbours whilst out	
		Whining or whimpering	
		Panting when leaving	
		Vomiting or drooling	
		Toileting (weeing or pooing) as leaving	
		Owners found damage around doorway on return	
		Preventing owners from leaving (e.g. blocking doorway)	
		Pacing, spinning, circling or tail chasing	
		Items other than toys found chewed or destroyed on return	48hr Behaviour call; Go to Q3.8
		Trying to get out through door with owners	
		Excitability or excessive greeting when owner returned	
		Problems getting back into the house on return	
		Other behaviour(s) when left (with Text box)	24-48hr Behaviour call; Go to Q3.8
		Text box for more information	
3.6a	Have you noticed that [Dog's name] has shown any of the following behaviours when [he/she] was left alone? **List out the behaviour signs in response options**	No signs noticed	Go to Q3.9
		Toileting (wee or poo) found on return	24hr Behaviour call; Go to Q3.9
		Owner heard dog barking as they were leaving	
		Owners heard howling on leaving	
		Chewing or scratching around doorway heard after owner left	
		Owners heard barking as they returned	
		Owners heard howling as they returned	
		Barking heard by neighbours whilst out	
		Howling heard by neighbours whilst out	
		Whining or whimpering	
		Panting when leaving	
		Vomiting or drooling	
		Toileting (weeing or pooing) as leaving	
		Owners found damage around doorway on return	
		Preventing owners from leaving (e.g. blocking doorway)	
		Pacing, spinning, circling or tail chasing	
		Items other than toys found chewed or destroyed on return	48hr Behaviour call; Go to Q3.8
		Trying to get out through door with owners	
		Excitability or excessive greeting when owner returned	
		Problems getting back into the house on return	
		Other behaviour(s) when left (with Text box)	

		Text box for more information	24-48hr Behaviour call; Go to Q3.8
3.7	Do you have any concerns about leaving your dog; would you like any advice on this?	Yes, some concerns, would like advice (with Text box)	48hr Behaviour call; Go to Q3.8
		Yes, some concerns but no advice needed	Go to Q3.8
		No concerns	
3.8	Have you noticed any other behaviours that you found a problem or were concerned about in the last month? If so, would you like any advice from our behaviour team?	Yes, other signs but don't need help	Go to Q3.9
		Yes, other signs and would like help	Go to Q3.11
		No, no other signs	Go to Section 4
3.9	Would you mind telling me which behaviours you have seen – just so I can make a note ?	Signs of aggression (Growling, baring teeth or wrinkling lips up, snapping, biting, nipping, lunging forward whilst barking, grabbing (e.g. grabbing lead), mouthing (e.g. at sleeves or arms) or standing very still and staring)	Go to Q3.10
		Problems when left alone or when separated from owner/family member e.g. barking, howling, whining, toileting, destruction	
		Difficult to walk on the lead e.g. pulling on the lead or refuses to walk	
		Toileting inside the home	
		Barks excessively e.g. in the garden	
		Barks at people or animals	
		Chews or destroys non-food items other than own toys	
		Mounts/humps person, dog, object (e.g. bed)	
		Follows people around house and/or trips people up	
		Won't get into or out of the car	
		Won't get off sofa/other furniture	
		Gets on furniture and we don't want them to	
		Steals food, scavenges or raids bins	
		Steals items other than own toys	
		Not giving up items including toys	
		Doesn't come back when called	
		Reacts to noises	
		Over active/doesn't settle down	
		Jumping up	
		Bothers another household dog	
		Bothers a non-household dog	
		Chases other dogs	
		Chases household cats or other pets	
		Chases local cats or wildlife	
		Chases joggers	
		Chases bikes	
		Chases vehicles	
		Chases livestock or horses	
		Restless or vocalising in car	
		Cowers/hides/runs away from moving vehicles	
		Digging	
		Toileting issue outside of the home	
		Is sick or drools during travelling	
		Escaping	
		Spins in circles or chases tail repetitively	
		Paces a lot	
		Barks at inanimate object e.g. cars or reflections	
		Plays excessively/roughly with another dog	
		Uninterested in play/reluctant to play	
		Wants to play excessively e.g. with toys	
		Pawing at or licking people	
		Shies away from people e.g.runs away/hides	
		Runs away/hides from other dogs	

3.9 (cont.)		Seems fixated on or chases points of light or shadows	
		Stares at walls or 'nothing'	
		Seems unreactive	
		Stares at people	
		Won’t go up or down stairs	
		Other behaviours (with Text box)	
		Text box for more information	
		Rather not say	Go to Section 4
3.10	Are you sure you don’t need any help from us? I’m happy to ask a colleague to give you a call if it would be useful?	No, don't need help	Go to Section 4
		Yes, please arrange call	
3.11	That’s absolutely fine. Could you let me know what problem you’re having, and I can arrange a call back for you	Signs of aggression (Growling, baring teeth or wrinkling lips up, snapping, biting, nipping, lunging forward whilst barking, grabbing (e.g. grabbing lead), mouthing (e.g. at sleeves or arms) or standing very still and staring)	24hr Behaviour call; Go to Section 4
		Problems when left alone or when separated from owner/family member e.g. barking, howling, whining, toileting, destruction	
		Not giving up items including toys	
		Bothers another household dog	
		Seems fixated on/chases points of light/shadow	
		Stares at walls or 'nothing'	
		Spins in circles or chases tail repetitively	
		Chases household cats or other pets	
		Toileting inside the home	48hr Behaviour call; Go to Section 4
		Barks excessively e.g. in the garden	
		Won't get off sofa/other furniture	
		Reacts to noises	
		Over active/doesn't settle down	
		Jumping up	
		Bothers a non-household dog	
		Chases other dogs	
		Chases joggers	
		Chases bikes	
		Chases livestock or horses	
		Cowers/hides/runs away from moving vehicles	
		Escaping	
		Plays excessively/roughly with another dog	
		Shies away from people e.g.runs away/hides	
		Mounts/humps person, dog, object e.g. bed	
		Difficult to walk on the lead e.g. pulling on the lead or refuses to walk	72hr Behaviour call; Go to Section 4
		Barks at people or animals	
		Chews or destroys non-food items other than own toys	
		Follows people around house and/or trips people up	
		Won't get into or out of the car	
		Gets on furniture and we don't want them to	
		Steals food, scavenges or raids bins	
		Steals items other than own toys	
		Doesn't come back when called	
		Chases local cats or wildlife	
		Chases vehicles	
		Restless or vocalising in car	
		Digging	
		Toileting issue outside of the home	
		Is sick or drools during travelling	
		Paces a lot	
		Barks at inanimate object e.g. cars/reflection	
		Uninterested in play/reluctant to play	
		Wants to play excessively e.g. with toys	

		Pawing at or licking people	
		Runs away/hides from other dogs	
		Seems unreactive	
		Stares at people	
		Won’t go up or down stairs	
		Rather not say	
		Other behaviours (with Text box)	
		Text box for more information	

Section 4: Adoption Experience

Finally, I’m going to ask you a few questions about your experiences when adopting [Dog’s name].

4.1	Would you say overall, you’re happy that you decided to adopt [Dog’s name]?	Not sure, depends on his/her behaviour	Go to Q4.2
		Not sure, it depends on his/her health	
		Not sure, it depends on his/her behaviour and health	
		Not sure, too early to say	
		No, unhappy about adoption for non-behaviour/health reasons	Go to Q4.3
		Yes, happy to have adopted dog	Go to Q4.4a
		Other response (with Text box)	
4.2	Have we already discussed any concerns that are worrying you in this call? If no, would you like some help?	Yes, already discussed	Go to Q4.4a
		No, owner has behaviour/health concerns not discussed but doesn't want advice	
		No, owner has health concerns not discussed and does want advice	24hr Vet call; Text box for details; Go to Q4.4a
		No, owner has behaviour concerns not discussed and does want advice	48hr Behaviour call; Text box for details; Go to Q4.4a
		No, owner has behaviour/health concerns not discussed and does want advice	48hr Behaviour & Vet call; Text box for details; Go to Q4.4a
4.3	Would you mind telling me why you’re not happy about adopting [Dog’s name]?	Unhappy about the process	Text box for details; Go to Q4.4a
		Wanted a different dog	
		Unhappy about customer service	
		Unexpected challenges of having a dog	
		Other response	
		Impact on lifestyle	
		Family member allergic to pet	
		Dog not getting on with other pets	
4.4a	This is the last follow-up call that we have scheduled with you as part of our post adoption support, although you can continue to call us for advice in the future. As this is a new service, we would love to hear your feedback on how you found these calls, and any follow up support you received. Overall, how useful did you find our calls (those that you have had)?	Very helpful	Go to Q4.4.b
		Quite helpful	
		Neutral	
		Quite unhelpful	
		Very unhelpful	
		Don’t know/rather not say	
		Text box for more information	
4.4b	If you have received any behaviour support from us so far, would you say that your experience with the behaviour staff has been very positive, quite positive, neutral, quite negative or very negative?	Very positive	Go to Q4.5
		Quite positive	
		Neutral	
		Quite negative	
		Very negative	
		Don’t know/can't remember/rather not say	
		Not applicable (haven’t had support)	
		Text box for more information	
4.5	Thank you so much for speaking with me today, it is amazing to hear how [Dog’s name] is getting on. We really appreciate your time, and I hope the call has been useful for you. We’ll be back in touch in a couple of weeks. Please do call us back if you have any more worries in the meanwhile. Goodbye.		End Call
